# Prediction of Composite Supercapacitor Performance Through Combining Machine Learning with Novel Binder-Related Features

**DOI:** 10.3390/nano16080478

**Published:** 2026-04-17

**Authors:** Tianshun Gong, Weiyang Yu, Xiangfu Wang

**Affiliations:** 1 College of Electronic and Optical Engineering & College of Flexible Electronics (Future Technology), Nanjing University of Posts and Telecommunications, Nanjing 210023, China; b23020625@njupt.edu.cn; 2 School of Physics and Electronic Information, Henan Polytechnic University, Jiaozuo 454003, China

**Keywords:** electrochemical performance, composite supercapacitor, machine learning, interpretability

## Abstract

The development of high-performance composite supercapacitors remains challenging because the specific capacitance of composite electrodes is jointly governed by electronic percolation, ion accessibility, and interfacial contact, all of which are strongly affected by the balance among active materials, conductive agents, and binders. Traditional equivalent circuit modeling and empirical trial-and-error methods are often inadequate for describing these non-linear relationships and optimizing electrode design. To address this limitation, we establish a physics-guided and interpretable machine learning (ML) framework for predicting the specific capacitance of composite electrodes. Unlike traditional methods that rely on macroscopic mass fractions, our approach constructs a feature space comprising ten descriptors, including two newly introduced binder-related proxy descriptors—Binder-to-Conductive Ratio (BCR) and Specific Binder Loading (SBL)—to better represent the influence of binder content. By systematically evaluating 17 machine learning algorithms on a high-fidelity dataset, we identify the XGBoost model, optimized via Bayesian optimization, as the best predictor, achieving a coefficient of determination (R^2^) of 0.981 and a low mean absolute percentage error (MAPE) of 14.49%. Importantly, interpretability analysis using Shapley Additive Explanations (SHAP) provides physically interpretable statistical insights, revealing that high BCR suppresses specific capacitance through an insulating barrier effect, whereas lattice distortion in the filler material promotes ion transport. This study offers a robust, data-driven framework for optimizing composite electrode performance, demonstrating the potential of interpretable ML models for the rational design of advanced energy-storage materials.

## 1. Introduction

Composite supercapacitors are advanced energy storage devices that combine the high power density of electrochemical capacitors with the energy storage capabilities of pseudocapacitive materials [1,2,3]. They are widely used in fast-charging and high-power energy-storage scenarios, including regenerative braking in electric vehicles, short-term grid power buffering, and peak-power support in portable electronics [4,5,6,7]. In these applications, specific capacitance is a key performance indicator because it directly reflects the charge-storage capability of the electrode and strongly influences the achievable energy density under practical operating conditions. A higher specific capacitance generally enables greater energy storage within limited mass and volume, whereas insufficient capacitance restricts device efficiency and application potential. Therefore, improving and accurately predicting the specific capacitance of composite electrodes is essential for the rational design of advanced supercapacitors.

Traditional approaches, such as equivalent circuit modeling and empirical trial-and-error optimization, have been widely used to evaluate the electrochemical behavior of supercapacitors [8,9]. However, these methods often oversimplify the highly coupled and nonlinear interactions among active materials, conductive additives, and binders in composite electrodes, making them insufficient for accurately predicting specific capacitance across diverse electrode formulations. In parallel, machine learning (ML) has rapidly emerged as a practical tool in materials research, particularly for predicting the performance of energy storage devices [10,11]. For example, Siraprapha Deebansok and colleagues used supervised machine learning to propose a “capacitance tendency” criterion to determine whether a material’s electrochemical behavior is more battery-like or pseudocapacitive [12]. Yuxuan Sun and colleagues employed three machine learning models—Random Forest (RF), Gradient Boosting Regression (GBR), and Extreme Trees Regression (ETR)—to establish quantitative models linking the preparation parameters of activated biochar to its energy storage properties [13]. Weigert and colleagues used fully connected neural networks to predict the cycle life of battery–supercapacitor hybrid electric vehicles and explore the main factors affecting supercapacitor aging [14]. These approaches have advanced our understanding of the electrochemical properties of supercapacitors and other energy storage materials, paving the way for more efficient material design and performance prediction [15]. However, for composite electrodes, predicting specific capacitance remains challenging. Most existing studies rely on material categories, preparation parameters, or macroscopic composition descriptors, while insufficiently capturing the interfacial competition among binder, conductive components, and active phases. As a result, the physical factors governing electronic percolation, ionic accessibility, and binder-induced blocking effects are often underrepresented in current predictive models. This limitation motivates the development of a feature framework that can better describe binder-related interfacial regulation in composite electrodes.

To overcome the limitations of traditional trial-and-error and material-centric studies, this work establishes an interpretable machine learning framework for composite supercapacitor electrodes. Rather than relying on coarse macroscopic descriptors, we construct a physically motivated feature space to capture multiscale factors governing electrode performance. By introducing two binder-related proxy descriptors—Binder-to-Conductive Ratio (BCR) and Specific Binder Loading (SBL)—we aim to more accurately capture the complex interfacial effects between the binder and conductive materials. These descriptors are integrated with intrinsic crystallographic properties (lattice constant and strain), processing history (drying temperature and time), microstructural attributes (pore type), and electrochemical testing conditions (electrolyte, current density, and voltage window). Through systematic benchmarking of multiple algorithms, an optimized XGBoost model was identified as the most robust predictor. More importantly, interpretability analysis based on Shapley Additive Explanations (SHAP) reveals that the interfacial regulation captured by BCR and SBL plays a dominant role in determining specific capacitance, often surpassing the influence of external testing conditions. Collectively, this framework provides a data-driven basis for interface-aware electrode design, while remaining distinct from a fully first-principles physical model.

## 2. Methods

### 2.1. Machine Learning in Materials Science

Machine learning aims to learn the mapping relationship f:X→Y between the input space X and output space Y through algorithms, with the core goal of minimizing the discrepancy between the model’s predicted values and actual values [16,17]. Mathematically, it can be expressed as follows: given the dataset $D={\left(x_{i},y_{i}\right)}_{i=1}^{N}$, by optimizing the model parameters θ, the loss function L(y,f(x;θ)) is minimized, which can be formulated as:

(1) $$\hat{\theta }=\arg\;\underset{\theta }{\min}\;\frac{1}{N}\sum_{i=1}^{N}\;L(y_{i},f(x_{i};\theta ))$$

Here, L(⋅) is used to measure prediction error, and $f\left(x;\theta \right)$ represents the parameterized model (e.g., linear models or neural networks). This optimization process reflects the core concept of machine learning in adaptively extracting patterns from data.

**Figure 1:**
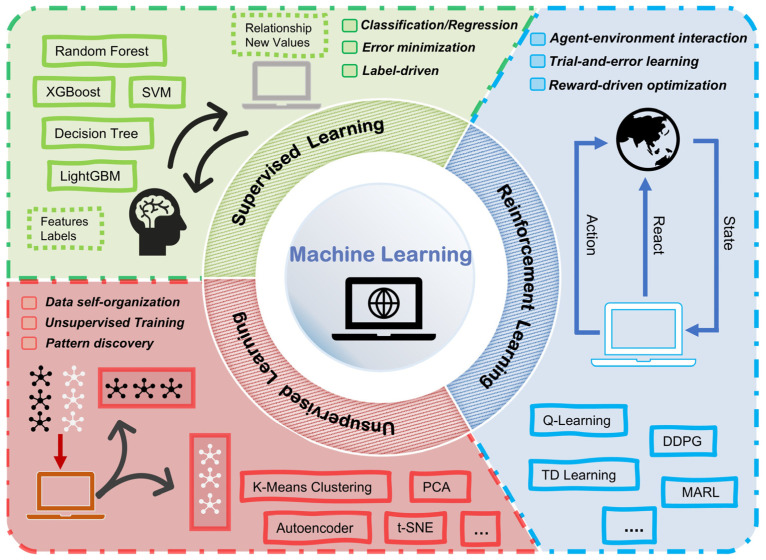
Common machine learning classifications and algorithms.

Machine learning models can be categorized into three fundamental paradigms (Figure 1) based on the learning mechanism: supervised learning, unsupervised learning, and reinforcement learning [18,19]. Supervised learning involves learning a mapping function from a labeled dataset $D=(x_{i},y_{i})$, where the goal is to minimize the difference between predicted values and true labels. Common tasks include regression and classification, with popular algorithms such as Linear Regression [20], Random Forests [21], and Decision Trees [22]. In contrast, unsupervised learning aims to identify underlying patterns in unlabeled data $D=x_{i}$, such as discovering clusters or learning the data’s probability distribution. It is often used for tasks like clustering and dimensionality reduction, with well-known algorithms like K-Means [23], principal component analysis (PCA) [24], and t-SNE [25]. Reinforcement learning involves an agent interacting with an environment to maximize a cumulative reward over time. Typical algorithms include Q-Learning [26], temporal difference learning (TD Learning) [27], and deep deterministic policy gradient (DDPG) [28].

We developed a physics-informed ML framework tailored for composite electrodes. Unsupervised clustering combined with stratified statistical analysis was first employed to ensure data robustness. Subsequently, multiple regression models were benchmarked to capture the intrinsically non-linear interactions within the composite system. Tree-based ensemble methods were found to be particularly effective in handling feature heterogeneity and interaction effects. Importantly, Shapley Additive Explanations (SHAP) were integrated to provide physically interpretable insights, enabling quantitative assessment of how interfacial regulation governs electrochemical performance. Detailed mathematical formulations of the algorithms are provided in the Supplementary Materials.

### 2.2. Charge Storage Mechanisms and Interfacial Regulation in Composite Supercapacitors

**Figure 2:**
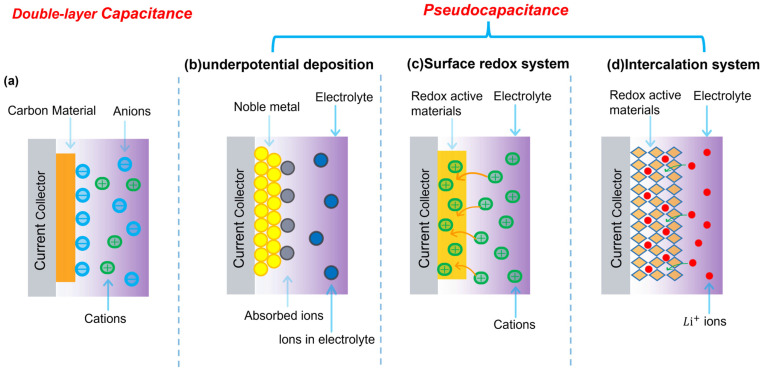
Schematic illustration of charge storage mechanisms of composite supercapacitors. (**a**) Electric double-layer capacitance (EDLC), primarily contributed by the conductive polymer-filler network; (**b**–**d**) Pseudocapacitive mechanisms including underpotential deposition, surface redox reactions, and intercalation.

Based on charge storage mechanisms, supercapacitors are generally categorized into electrochemical double-layer capacitors (EDLCs) and pseudocapacitors [29,30]. EDLCs store energy via electrostatic charge separation caused by the reversible adsorption of electrolyte ions at the electrode–electrolyte interface. As illustrated in Figure 2a, upon applying an external voltage, electrolyte ions migrate to form an electrochemical double layer along the surface of the conductive composite network. While EDLCs offer rapid charge/discharge capabilities and excellent cycle stability, their capacitance is primarily governed by the effective specific surface area (A)—a parameter critically regulated by binder distribution and surface coverage within the porous electrode framework [31]. The capacitance equation is:

(2) $$\;C=\frac{\varepsilon_{0}\varepsilon_{r}A}{d}$$

where $\varepsilon_{0}$ is the vacuum dielectric constant, $\varepsilon_{r}$ is the relative dielectric constant, d represents the EDL thickness, and A denotes the effective interfacial contact area.

In contrast, pseudocapacitors store charge via fast and reversible Faradaic redox reactions driven by thermodynamic potentials [32,33]. These processes include underpotential deposition (Figure 2b), surface redox reactions (Figure 2c), and intercalation pseudocapacitance (Figure 2d) [34]. Among them, intercalation pseudocapacitance is intrinsically governed by the crystallographic characteristics of the active phase, particularly lattice parameters and lattice distortion, which dictate ion diffusion pathways and kinetic energy barriers. In such materials, ions migrate through well-defined crystallographic channels within the host lattice rather than being confined to surface sites. Accordingly, parameters such as interlayer spacing and bottleneck size determine diffusion accessibility and activation energy for ion hopping [35]. Lattice distortion or strain changes the local coordination environment and electrostatic potential, thus affecting the energy landscape for ion transport [36,37,38]. Previous studies have shown that appropriate lattice expansion or tensile strain can effectively lower ion migration barriers and enable rapid, reversible intercalation without inducing sluggish phase transformations, a hallmark of intercalation pseudocapacitance [39]. Despite their high theoretical capacitance, pristine inorganic pseudocapacitive materials often suffer from limited electronic conductivity and structural instability during cycling [40]. In composite electrodes, these limitations are alleviated by conductive percolation networks; however, this introduces an interfacial trade-off, as binder-mediated mechanical cohesion inherently competes with electronic transport. Therefore, precise regulation of interfacial topology is required to maintain percolation pathways while ensuring structural stability. In the present machine-learning framework, the redox activity of pseudocapacitive materials is not introduced as a directly measured single variable. Instead, it is represented through a group of physics-informed descriptors that are closely associated with Faradaic charge-storage behavior, including lattice constant, lattice distortion, electrolyte environment, and voltage window. These descriptors collectively reflect ion diffusion accessibility, local coordination changes, and the thermodynamic/kinetic conditions under which reversible redox reactions proceed.

Overall, the specific capacitance of composite supercapacitors is governed by two coupled processes: charge accumulation at the electrode–electrolyte interface and ion transport within the porous electrode matrix. Performance is thus determined by a hierarchy of factors spanning multiple length scales. At the atomic scale, lattice constants and lattice distortion of the active phase control intrinsic charge storage capacity and ion migration barriers [41]. At the microstructural scale, pore architecture and accessible surface area dictate ion transport efficiency, with hierarchical porosity playing a critical role in maximizing active surface exposure [42]. At the macroscopic scale, electrode formulation—particularly the balance between binder and conductive components—regulates effective electronic conductivity and internal resistance. Finally, electrolyte properties and testing conditions impose thermodynamic and kinetic constraints. Consequently, optimizing specific capacitance requires an integrated framework that links crystallographic features, interfacial regulation, and external operating conditions. From the perspective of configuration analysis, the present ML framework can be interpreted as a unified model applicable to both EDLC-dominated and pseudocapacitive composite electrodes, while the dominant descriptors differ between the two regimes. For EDLC-like systems, the key controlling factors are ion-accessible interfacial area and pore accessibility; therefore, binder-related descriptors such as SBL and BCR mainly reflect pore blockage, effective surface exposure, and conductive network continuity. In pseudocapacitive systems, the dominant factors shift toward redox-active phase characteristics and ion transport within or near the active lattice; accordingly, Lattice_A, Strain, electrolyte type, and voltage window become more directly associated with performance, while BCR and SBL continue to regulate the utilization efficiency of redox-active sites through interfacial transport constraints. This unified perspective provides the basis for applying a data-driven framework to predict and optimize the performance of composite supercapacitors across different charge-storage regimes.

### 2.3. Feature Engineering and Machine Learning Framework for Composite Supercapacitors

Constructing a high-quality dataset is a prerequisite for developing reliable machine-learning models, as data fidelity directly governs generalization performance [43]. To ensure representative coverage of composite electrode systems, a systematic literature survey spanning 2013–2025 was conducted using major academic databases, including Scopus, Web of Science, ScienceDirect, and IEEE Xplore. A strict zero-missing-value criterion was applied: only studies reporting complete synthesis and testing parameters were retained, yielding a curated dataset of 191 high-fidelity samples (Supplementary Materials, Table S1). This dataset encompasses a broad range of heterogeneous composite architectures, covering diverse inorganic active phases and interfacial binding environments, thereby enabling the exploration of structure–performance relationships across diverse reported composite systems within the scope of the current literature-derived dataset.

Guided by the multi-scale framework outlined in Section 2.2, a physically motivated feature space comprising ten descriptors was constructed to decouple interfacial regulation from intrinsic material activity. At the atomic scale, intrinsic crystallographic descriptors—including lattice constant (Lattice_A) and lattice distortion (Strain)—were selected to represent the theoretical charge-storage capability of the active phase. To explicitly capture interfacial competition within composite electrodes, two interfacially motivated proxy descriptors were introduced. The Binder-to-Conductive Ratio (BCR) quantitatively characterizes the balance between insulating binder content and the electronic percolation network:

(3) $$BCR=\frac{m_{binder}}{m_{conductive}}$$

In parallel, the Specific Binder Loading (SBL) estimates effective surface coverage of the binder on the porous framework, serving as a proxy for ion accessibility and binder-induced pore blockage:

(4) $$SBL=\frac{m_{binder}}{m_{active}\times SSA}$$

where m represents mass and SSA denotes the specific surface area.

Microstructural and processing parameters were further incorporated, including pore type (categorized as Mesoporous or Hierarchical) and drying history ($T_{dry}$, $t_{dry}$). Finally, the external testing environment was standardized by incorporating electrolyte type (Neutral/Alkaline), Current Density ($J_{load}$), and voltage window ($V_{win}$) as kinetic and thermodynamic boundary conditions. The specific capacitance ($C_{s}$, F/g) was set as the target output variable, allowing the framework to learn the complex non-linear mappings between crystallographic features, interfacial descriptors and the final electrochemical performance. From a mechanistic perspective, the descriptors used in this study can be grouped according to the processes they represent. Among them, Lattice_A and Strain are closely related to the intrinsic redox-active behavior of pseudocapacitive phases, because they affect ion transport pathways and the energetic landscape of reversible Faradaic reactions. Electrolyte type and voltage window further define the external conditions under which such redox processes can be activated and sustained. Therefore, the machine-learning model interprets redox activity through the combined statistical effects of these physically meaningful descriptors.

Following data collection, a multi-step preprocessing strategy was implemented to ensure statistical robustness. Given the heterogeneity of the composite systems, an unsupervised K-means clustering algorithm was first applied to partition the dataset into distinct subgroups based on material characteristics. Subsequently, box plot analysis was utilized within these subgroups to identify and exclude statistical anomalies, ensuring that the model captures generalizable physical laws rather than fitting to extreme outliers. The K-means clustering and subgroup-wise box-plot analysis were used only during the raw-data curation stage to identify anomalous records in the literature-derived dataset before model development, rather than as part of the predictive learning pipeline. To eliminate dimensional heterogeneity across continuous features with vastly different magnitudes, the Min-Max normalization technique was employed. This linearly maps all continuous input variables to the $\left[0,1\right]$ range, accelerating the convergence of gradient descent algorithms. The normalization formula is defined as:

(5) $$X_{\mathrm{norm}}=\frac{X-X_{\min}}{X_{\max}-X_{\min}}$$

where $X_{\mathrm{norm}}$ is the normalized data, X is the original data, and $X_{\min}$ and $X_{\max}$ represent the minimum and maximum values in the dataset, respectively.

**Figure 3:**
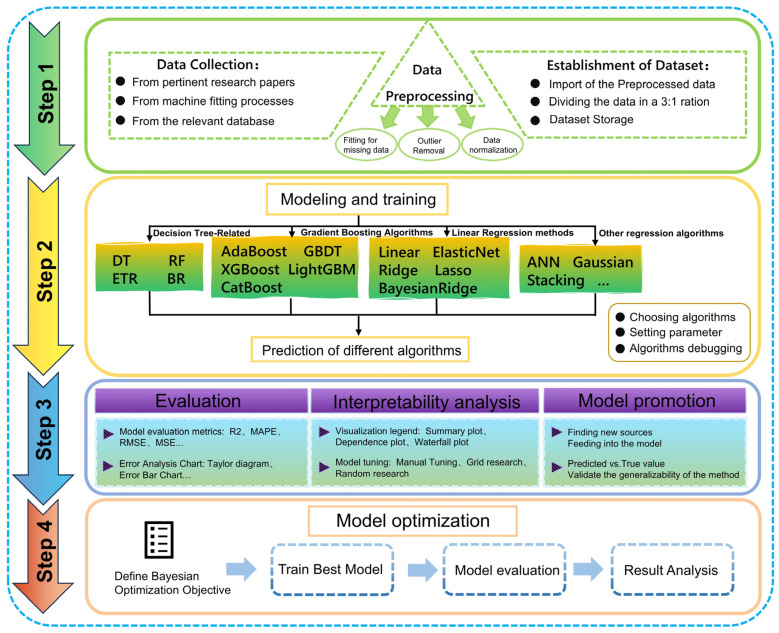
Schematic workflow of the physics-informed machine learning framework developed for composite supercapacitors.

To evaluate the predictive capability of the proposed physics-informed feature space, a comprehensive machine learning workflow was established, as illustrated in Figure 3. Consistent with standard protocols in materials informatics, the manually curated dataset of 191 high-fidelity samples was partitioned using a 3:1 ratio, yielding a training set of 143 samples and a testing set of 48 samples, and the corresponding random-seed setting was fixed to ensure reproducibility. To avoid information leakage, all model-dependent preprocessing and optimization steps were conducted using the training data only. Specifically, Min-Max normalization was fitted on the training set and then applied to the testing set using the same scaling parameters without refitting. Likewise, Bayesian optimization of the XGBoost hyperparameters was carried out exclusively on the training set, while the held-out test set was used only for final performance evaluation. Standard statistical metrics, including the Coefficient of Determination ($R^{2}$), Root Mean Square Error (RMSE), Mean Square Error (MSE), and Mean Absolute Percentage Error (MAPE), were employed to rigorously quantify the predictive accuracy and generalization capability of the framework. The detailed mathematical formulations for these metrics are provided in Table 1. In addition to the single held-out 3:1 train/test split, repeated k-fold cross-validation was performed to assess the stability of model performance against data partitioning. The mean and standard deviation of R^2^, MAPE, and RMSE across repeated runs were reported to quantify statistical variation in Supplementary Materials, Table S4. Beyond predictive accuracy, interpretability was emphasized to extract physically meaningful insights. Accordingly, Shapley Additive Explanations (SHAP) [44] were employed to elucidate how individual descriptors contribute to model outputs. Rather than serving solely as a feature-ranking tool, SHAP enables quantitative interpretation of interfacial competition by resolving the respective contributions of BCR and SBL, thereby linking binder-mediated regulation to specific capacitance and reinforcing the physical relevance of the proposed framework. To improve reproducibility, the full curated dataset used for model development and the independent external validation dataset are provided in the Supplementary Materials (Tables S1 and S2, respectively). In addition, the optimized hyperparameters of the final XGBoost model and the hyperparameter settings of the main baseline models are summarized in Tables S5 and S6, respectively.

## 3. Results and Discussion

### 3.1. Data Preprocessing Analysis

**Figure 4:**
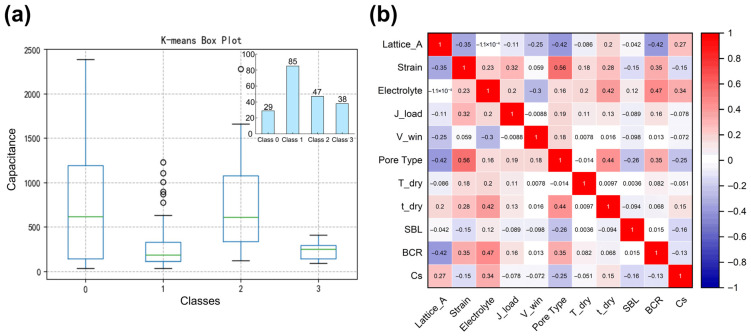
Data preprocessing and feature correlation analysis. (**a**) K-means clustering box plot (**b**) Pearson correlation heatmap used to screen for multicollinearity.

As visualized in Figure 4a, unsupervised K-means clustering analysis was employed to partition the heterogeneous dataset into four statistically distinct subgroups. This stratification confirms that the input feature space exhibits complex, non-uniform distributional patterns that cannot be effectively captured by a single global distribution. Crucially, this cluster-based segmentation provided the necessary local context for precision noise reduction. As demonstrated in the box plot analysis, applying stratified outlier detection within each specific subgroup prevented the erroneous exclusion of valid data points, which might appear extreme globally but remain physically valid within their specific local context. Consequently, this stratified preprocessing strategy rigorously filtered out experimental noise and statistical artifacts, ultimately yielding a finalized dataset of 191 high-fidelity samples. This rigorous data hygiene ensures that the subsequent machine learning models are trained on reliable and representative data.

Subsequent to outlier removal, a Pearson correlation heatmap was generated (Figure 4b) to systematically screen for multicollinearity among the ten input descriptors. As evidenced by the heatmap, the majority of pairwise correlation coefficients are confined below a threshold of 0.6, indicating that the selected feature space is robust and contains independent physical information with minimal redundancy. Crucially, the correlation between the two newly engineered interfacial descriptors—BCR and SBL—is negligible. This statistical orthogonality confirms that our framework successfully decouples the distinct regulatory roles of the binder: BCR specifically characterizes the electronic percolation competition, while SBL independently governs ionic accessibility, operating without statistical overlap. Furthermore, the relatively weak linear correlations observed between individual features and specific capacitance ($C_{s}$) imply that the structure–property relationships in these composite systems are governed by complex non-linear interactions rather than simple linear dependencies. This observation provides a compelling justification for employing advanced ensemble learning algorithms, which excel at capturing high-dimensional interaction effects that linear baselines cannot resolve.

### 3.2. Performance Comparison of Linear Regression and Decision Tree-Based Models

**Figure 5:**
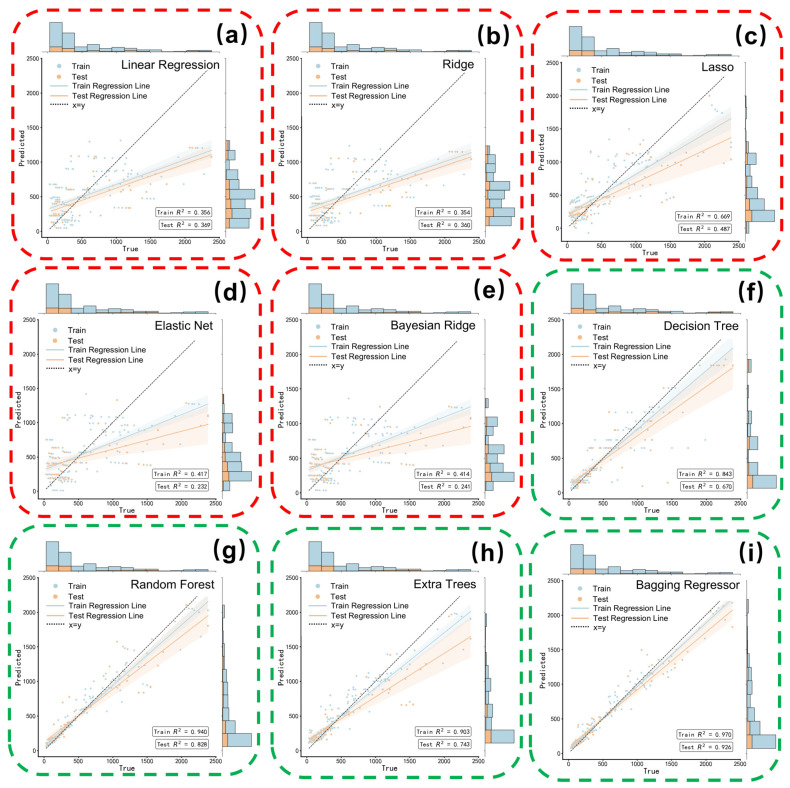
Predicted Specific Capacitance and Actual Values Based on Training and Test Datasets: Linear Regression Models: (**a**) Linear Regression; (**b**) Ridge Regression; (**c**) Lasso Regression; (**d**) Elastic Net; (**e**) Bayesian Ridge Regression. Decision Tree-Based Models; (**f**) Decision Tree; (**g**) Random Forest; (**h**) Extra Trees Regressor; (**i**) Bagging Regressor.

Figure 5 compares the predictive performance of linear regression and decision tree-based models using parity plots, where proximity to the diagonal line reflects prediction accuracy, and marginal distributions reveal potential bias. Quantitative evaluation metrics are summarized in Table 2.

Linear regression models consistently fail to capture the variance in specific capacitance, with all variants exhibiting limited predictive power. Even the best-performing linear model, Lasso regression, achieved an R^2^ of only 0.49, indicating that capacitance cannot be described as a linear superposition of individual descriptors. This limitation reflects the inherently non-linear nature of composite electrodes, where interfacial competition between insulation and electronic percolation governs performance and cannot be resolved by linear approximations.

In contrast, decision tree-based models demonstrate substantially improved predictive capability by capturing non-linear feature interactions. Although a single decision tree suffers from overfitting, ensemble approaches effectively mitigate this issue. Random Forest yields a robust fit (R^2^ ≈ 0.83), while the Bagging Regressor further improves accuracy (R^2^ ≈ 0.93), highlighting the importance of ensemble learning for handling the heterogeneity and interaction-dominated behavior of composite electrode systems. These results motivate the use of more advanced boosting strategies to further optimize the bias–variance balance.

### 3.3. Gradient Boosting Algorithms and Other Common Models

**Figure 6:**
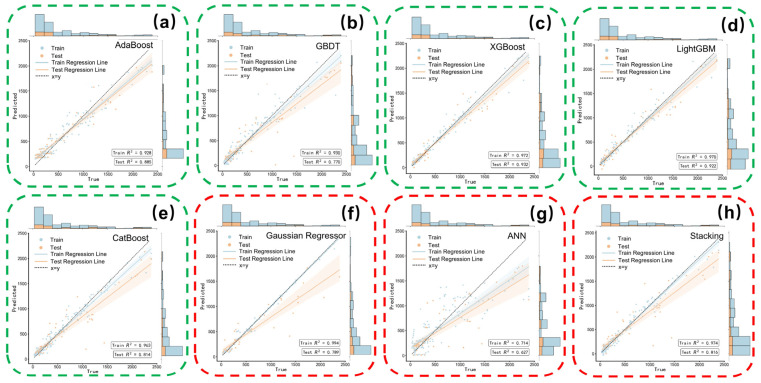
Predicted Specific Capacitance and Actual Values Based on Training and Test Datasets: Gradient Boosting Models: (**a**) AdaBoost; (**b**) GBDT; (**c**) XGBoost; (**d**) LightGBM; (**e**) CatBoost. Other Common Models; (**f**) Gaussian Process Regressor; (**g**) Artificial Neural Network (ANN); (**h**) Stacking Ensemble Algorithm.

Among the evaluated architectures, gradient boosting algorithms demonstrated a decisive advantage over linear and single-tree models, as detailed in Figure 6 and Table 3. Most notably, under the present train/test split and evaluation setting, XGBoost showed the best overall predictive performance among the evaluated models, achieving the highest $R^{2}$ of 0.9323 and a remarkably low MAPE of 26.05% on the test set. Its success is attributed to advanced regularization terms and parallelized tree construction, which effectively modeled the non-linear feature interactions while maintaining a balanced bias-variance trade-off. LightGBM also performed robustly with an $R^{2}$ of 0.9218, striking a good balance between efficiency and accuracy. In contrast, while AdaBoost exhibited the best stability (Test/Train MSE ratio of 2.11), its high MAPE indicated a compromise in precision. CatBoost, despite its popularity in other domains, suffered from severe overfitting in this task, likely due to its optimization focus on categorical features rather than the continuous physicochemical descriptors used here.

Regarding other architectures, Gaussian Process Regression (GPR) achieved a moderate $R^{2}$ of 0.7891 but exhibited high variance on the testing set. Notably, the Artificial Neural Network (ANN) significantly underperformed, with an $R^{2}$ of only 0.6273. This poor convergence highlights a known limitation in materials informatics: deep learning models struggle to extract features effectively from limited datasets (N<200), whereas ensemble tree methods demonstrate superior data efficiency. Similarly, while Stacking improved upon single models, it failed to surpass the optimized gradient boosting baselines.

To further assess whether the observed model ranking was sensitive to a specific train/test split, repeated cross-validation was additionally conducted for the top-performing models. As summarized in Supplementary Materials, Table S4, XGBoost remained the best overall performer on average, while the associated standard deviations provide an estimate of statistical variation across repeated resampling runs.

### 3.4. Validation on Novel Composites

To provide a preliminary assessment of external transferability, we further evaluated the five top-performing algorithms (XGBoost, AdaBoost, Bagging Regressor, LightGBM, and Random Forest) on an independent validation set. This set comprises four newly reported inorganic-based composite electrode materials derived from the recent literature (detailed in Supplementary Materials, Table S2). Although the sample size is limited, these unseen materials offer an initial out-of-sample check of model behavior beyond the original training dataset. It is acknowledged that minor metric fluctuations are statistically anticipated when transitioning to novel material systems, particularly given the finite sample size and the stochastic nature of data partitioning. However, such a stress test is essential for assessing true model robustness beyond the training distribution. The corresponding predictive results are provided in Supplementary Materials, Table S3.

**Figure 7:**
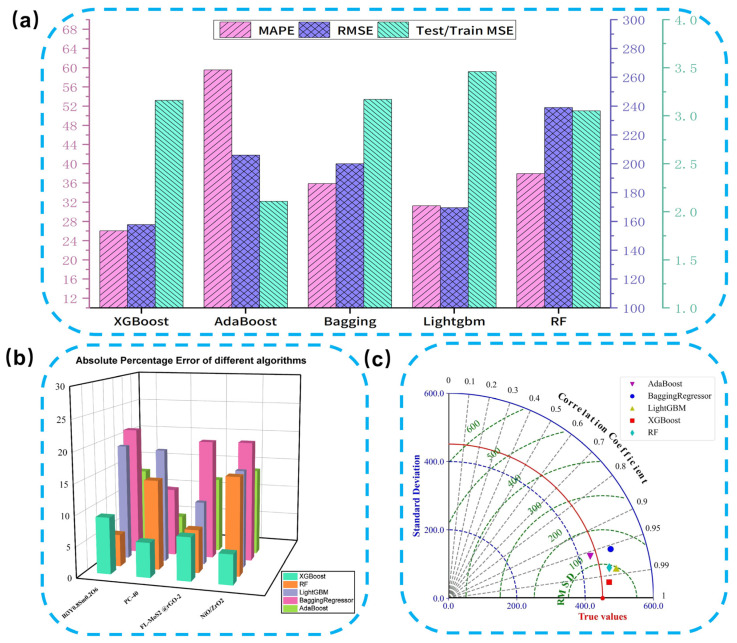
(**a**) Bar chart of MAE, RMSE, and Test/Train MSE values for different models; (**b**) Comparison of absolute percentage error of different algorithms for Materials Suitable for Supercapacitors Discovered in Recent Studies; (**c**) Taylor Diagram of Different Prediction Algorithms.

Figure 7a shows a bar chart displaying the MAPE, RMSE, and the Test/Train MSE ratio of different algorithms. XGBoost performed the best in terms of MAPE, with the smallest error and minimal fluctuation in prediction errors across samples, demonstrating strong stability. In contrast, AdaBoost showed greater error fluctuations, with a MAPE of 59.87 and poor stability. The error performance of Bagging Regressor, LightGBM, and Random Forest was between that of XGBoost and AdaBoost, with LightGBM showing more concentrated error distribution, performing slightly better than Bagging Regressor and Random Forest. Figure 7b features a 3D bar chart further illustrating the error distribution of different models. XGBoost had the smallest absolute percentage error, with minimal error fluctuations across samples, indicating strong accuracy and stability on the new dataset. AdaBoost, however, exhibited inferior performance across most samples, with greater error fluctuations, leading to worse overall performance.

The Taylor plot in Figure 7c further supports these findings, with XGBoost achieving the highest correlation coefficient and the smallest RMSE, demonstrating a strong linear relationship between predicted and actual values. While LightGBM and Random Forest also show good performance, their correlation coefficients are slightly lower and their RMSE is higher. AdaBoost, with the lowest correlation coefficient and higher RMSE, ranks the worst in terms of overall performance. Overall, within this preliminary external validation, XGBoost showed the most favorable balance of prediction accuracy and error stability among the evaluated models, therefore identified as the optimal model for predicting the specific capacitance of composite supercapacitors.

### 3.5. Model Interpretability Analysis

**Figure 8:**
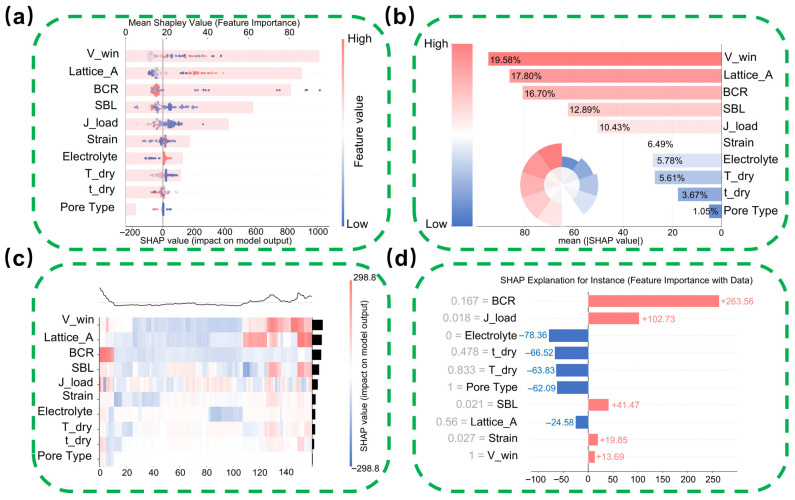
Multi-dimensional visualization of feature contributions using the SHAP framework. (**a**) SHAP summary bee-swarm plot showing the polarity and magnitude of feature impact on model output; (**b**) Bar chart ranking the top features by mean absolute SHAP value, highlighting the significance of BCR and SBL; (**c**) SHAP heatmap displaying the variation in feature effects across all samples; (**d**) Local explanation waterfall plot illustrating the decision path for a specific sample prediction.

To elucidate how material and interface-related descriptors govern the XGBoost predictions, the SHAP framework was employed to quantify feature contributions. As shown in Figure 8b, features are ranked by their mean absolute SHAP values. Beyond the external testing condition (V_win), which primarily constrains the achievable energy window, the crystallographic parameter Lattice_A and the interfacial descriptor BCR emerge as the dominant intrinsic contributors to specific capacitance. Notably, BCR surpasses conventional processing parameters such as drying temperature (T_dry), highlighting the critical role of interfacial competition between the insulating polymer phase and the conductive network in determining electrochemical performance. The high ranking of SBL further underscores the necessity of explicitly decoupling binder-related interfacial effects from active material loading during feature construction.

A more detailed material-level interpretation is obtained from the SHAP summary plot in Figure 8a, where the distribution of feature contributions reflects distinct interfacial and structural effects. For BCR, high values are predominantly associated with negative SHAP contributions, whereas low values cluster on the positive side, indicating that excessive binder relative to the conductive phase statistically suppresses specific capacitance. This trend is statistically consistent with an interfacial blocking effect, in which an increased polymer presence disrupts electronic percolation pathways. SBL exhibits a broader and non-monotonic distribution, suggesting that although a minimum binder coverage is necessary to maintain electrode integrity, excessive or uneven surface coverage can hinder ion accessibility. In contrast, Lattice_A shows a predominantly positive contribution, with larger lattice constants statistically favoring higher capacitance, suggesting that lattice expansion in the inorganic phase facilitates ion transport, in agreement with reported pseudocapacitive behavior. These results suggest that the model captures redox-related contributions in an interpretable manner. In particular, the positive SHAP contribution of Lattice_A indicates that crystallographic environments favoring ion transport are statistically associated with enhanced pseudocapacitive charge storage. Thus, the ML model does not directly “observe” redox reactions, but it infers their contribution from structure- and environment-dependent descriptors that govern the accessibility and reversibility of Faradaic processes.

Beyond linear trends, the SHAP heatmap in Figure 8c reveals how combinations of structural and interfacial descriptors collectively shape electrochemical performance across the dataset. High-capacitance regions are preferentially associated with the coexistence of a broad operating voltage window (V_win) and optimized Lattice_A values, indicating a synergistic interplay between electrochemical stability limits and intrinsic crystallographic features. At the individual electrode level, the SHAP waterfall plot in Figure 8d illustrates how a balanced interfacial configuration contributes to enhanced performance. In the representative high-capacitance sample, an optimized BCR of 0.167 provides a dominant positive contribution to the predicted capacitance, highlighting the critical role of tuning the competition between electronic conductivity and mechanical binding. This localized analysis reinforces that high performance emerges from coordinated regulation of interfacial and structural parameters rather than from any single descriptor in isolation. In this sense, the SHAP analysis also provides a configuration-level interpretation of the unified model: descriptors such as SBL and BCR are more closely linked to EDLC-related interfacial accessibility and conduction continuity, whereas Lattice_A, Strain, electrolyte, and voltage window are more strongly associated with pseudocapacitive redox/intercalation behavior. This distinction helps explain how the same ML framework can accommodate different charge-storage regimes within composite supercapacitors.

### 3.6. Ablation Study of BCR and SBL Descriptors

As the performance and interpretability of the original models have been discussed in the previous sections, this section focuses specifically on evaluating the independent contribution of the composite descriptors BCR and SBL through an ablation study. Since both descriptors are constructed from underlying formulation and processing parameters, it is essential to assess whether they contribute non-redundant predictive and interpretive information, rather than to claim direct proof of microscopic causality.

To this end, the five top-performing models (XGBoost, AdaBoost, Bagging Regressor, LightGBM, and Random Forest) were retrained after removing BCR and SBL from the input feature set, while all other model configurations and training procedures were kept unchanged. The quantitative performance of the ablated models is summarized in Table 4. Across all models, the removal of BCR and SBL leads to a consistent degradation in predictive accuracy, as evidenced by reduced R^2^ values and increased MAPE and RMSE. Notably, the increase in MAPE ranges from 5.7% to 11.0%, indicating that the loss of these descriptors systematically compromises prediction precision rather than affecting only a specific learning algorithm.

**Figure 9:**
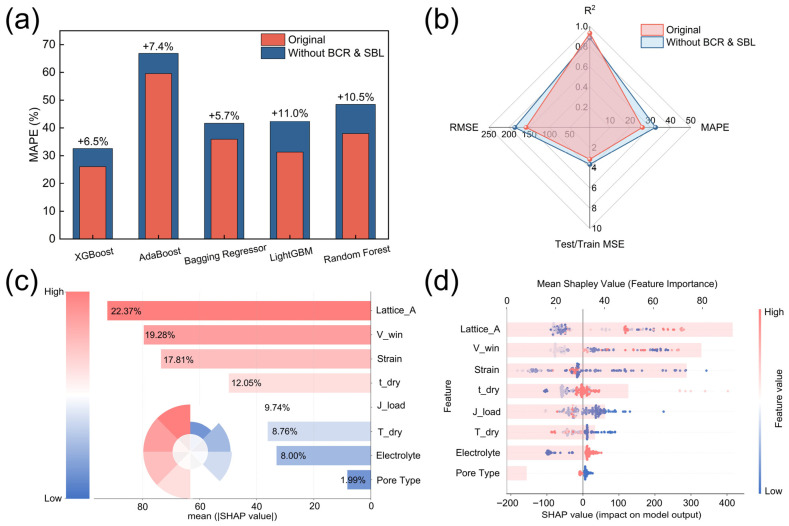
Ablation analysis evaluating the independent contribution of BCR and SBL descriptors. (**a**) Comparison of MAPE values for the five top-performing models before and after removing BCR and SBL; (**b**) Radar chart summarizing multi-metric performance of the XGBoost model under original and ablated feature settings; (**c**) Mean absolute SHAP value ranking after removal of BCR and SBL; (**d**) SHAP summary plot of the ablated model.

The performance degradation is further visualized in Figure 9a, where all five models exhibit higher MAPE values after ablation. Importantly, the relative ranking of model performance remains largely unchanged, with XGBoost still outperforming the other models. This observation suggests that the observed performance deterioration is primarily feature-driven rather than model-dependent. A holistic comparison using a radar chart for the XGBoost model is provided in Figure 9b. Although certain axes (MAPE, RMSE, and Test/Train MSE) show outward expansion after ablation due to their inverse performance semantics (lower values indicating better performance), this expansion corresponds to degraded accuracy and generalization stability. When interpreted together with the reduction in R^2^, the radar plot consistently reflects an overall decline in model performance upon removal of BCR and SBL, rather than any compensatory improvement.

To further elucidate how feature attribution changes after ablation, SHAP analysis was performed on the retrained models without BCR and SBL. The mean absolute SHAP value ranking of the remaining descriptors is shown in Figure 9c. In the absence of BCR and SBL, feature importance becomes more concentrated on intrinsic crystallographic parameters (e.g., *Lattice_A* and *Strain*) and external testing conditions (e.g., *V_win*), indicating a redistribution of explanatory weight toward a reduced subset of features. The corresponding SHAP summary plot of the ablated model (Figure 9d) reveals the sample-level impact of this redistribution. Compared with the more balanced attribution patterns observed in the original model (Section 3.5), the ablated model exhibits broader SHAP value dispersion for several remaining features, with increased extremes on both positive and negative sides. This behavior suggests that, after removal of BCR and SBL, the model becomes more sensitive to variations in individual descriptors, consistent with the observed increase in prediction error.

Overall, the ablation study demonstrates that BCR and SBL are not redundant descriptors that can be trivially replaced by reweighting the existing variables. Instead, their removal leads to simultaneous performance degradation and less stable feature attribution, indicating that these descriptors encode physically motivated, non-redundant information related to binder-regulated interfacial effects and insulation–percolation competition in composite electrodes.

### 3.7. Bayesian Optimization of the Model

Bayesian optimization is a global optimization method based on a Bayesian statistical framework [45]. By constructing a probabilistic model of the objective function, it efficiently finds the optimal solution. In machine learning, this method is commonly used to optimize hyperparameter combinations to improve model performance. In this study, Bayesian optimization was applied to further refine the hyperparameters of the XGBoost model to improve its predictive accuracy. The optimal hyperparameter combination obtained through optimization is as follows: n_estimators = 200, max_depth = 5, learning_rate = 0.11899, gamma = 1.14600, subsample = 0.64586, colsample_bytree = 0.62297, reg_lambda = 7.90319, reg_alpha = 7.39033. The detailed optimization process can be found in Figure S1 of the Supplementary Materials.

**Figure 10:**
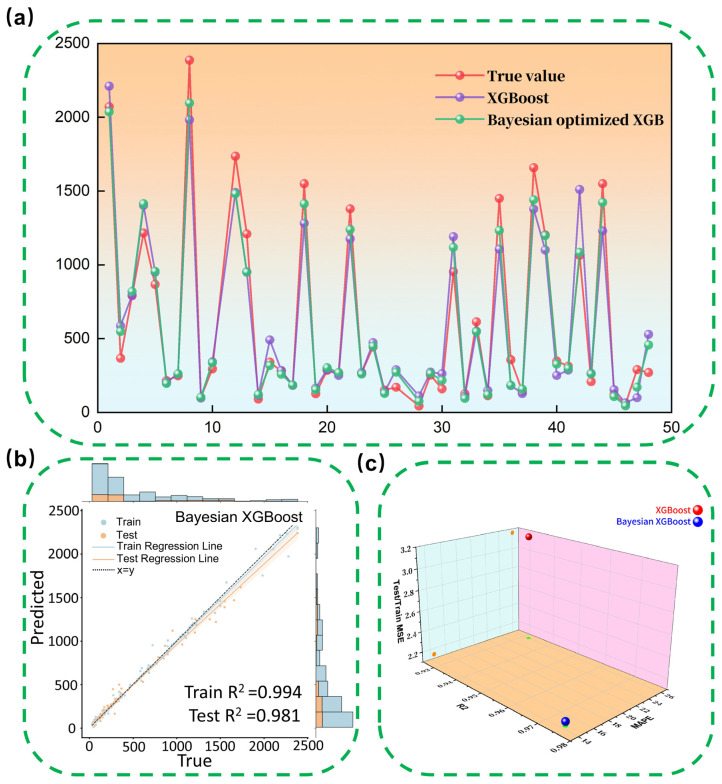
(**a**) Comparison Between Predicted and Actual Specific Capacitance Values of Supercapacitors Using XGBoost and Bayesian-Optimized XGBoost; (**b**) Bayesian-Optimized XGBoost algorithm for Predicted Specific Capacitance and Actual Values of Supercapacitors with Different Materials Based on Training and Test Datasets; (**c**) Relationship Between R^2^, MAPE, and Test/Train MSE for XGBoost and Bayesian-Optimized XGBoost.

Figure 10a compares the prediction results of XGBoost before and after Bayesian optimization. The *x*-axis represents the sample number, and the *y*-axis represents the specific capacitance values. The results show that the optimized XGBoost (green line) generates predictions that are much closer to the actual values (red line), especially for larger specific capacitance values, where the effect of the model optimization is particularly significant, with the prediction trend closely matching the actual values. In contrast, the unoptimized XGBoost (purple line) exhibits large prediction errors in certain regions (e.g., around sample numbers 20 and 40), demonstrating that Bayesian optimization substantially enhanced the model’s predictive accuracy.

Figure 10b shows the fitting curves of the optimized XGBoost model on both the training and testing sets, further validating the superior fitting performance of the optimized model. To comprehensively evaluate the optimization effect, Table 5 compares the XGBoost model with the Bayesian-optimized XGBoost model using metrics such as $R^{2}$, MAPE, RMSE, and Test/Train MSE. The results show that the Bayesian-optimized model outperforms the unoptimized model in $R^{2}$, MAPE, and RMSE. Significant reductions in both mean absolute percentage error and root mean square error demonstrate the optimization’s effectiveness. The improvement in Test/Train MSE further indicates that Bayesian optimization has enhanced the model’s generalization ability.

Figure 10c visually displays the changes in the correlation coefficient of XGBoost before and after Bayesian optimization through a 3D plot. The analysis shows that Bayesian optimization not only effectively reduced prediction errors but also improved the model’s ability to explain data fluctuations, making it more reliable for practical applications. By optimizing the hyperparameters, Bayesian optimization not only made the model better fit the training data, but also enhanced its adaptability to new datasets, providing strong support for efficient specific capacitance prediction of supercapacitors.

### 3.8. Challenges and Limitations

Despite the encouraging predictive performance, several challenges remain that are primarily rooted in materials data heterogeneity rather than model selection. High-quality datasets with standardized reporting protocols are still scarce, particularly for composite electrodes. Variations in binder molecular weight, solvent systems, and slurry preparation conditions are widespread across the literature and are often incompletely reported. Such inconsistencies introduce unavoidable uncertainty, limiting the reliable extraction of process–structure–property relationships. Addressing this limitation will require the development of community-level materials databases in which interfacial and processing parameters are systematically recorded alongside electrochemical metrics. Moreover, although the dataset was manually curated for completeness and data fidelity, the total sample size remains limited relative to the compositional and structural diversity of composite supercapacitor systems. Therefore, the present results should be interpreted as statistically supported within the current dataset scope, rather than as evidence of universal generalization across all composite electrode chemistries. We also note that the present study adopts a unified regression framework for heterogeneous composite systems rather than explicitly classifying each sample into a purely EDLC or purely pseudocapacitive regime. A more fine-grained regime-specific modeling strategy may be explored in future work when larger and more uniformly labeled datasets become available.

In addition, although this work incorporates intrinsic structural descriptors together with compositional metrics such as BCR and SBL, quantitatively resolving interfacial interactions remains a major challenge. The current descriptors are largely derived from static formulation parameters and therefore cannot fully capture the dynamic evolution of the polymer–electrode interface during cycling, including binder swelling, mechanical fatigue, or volumetric changes induced by electrolyte penetration. These time-dependent interfacial phenomena are difficult to encode into fixed features, highlighting the need for advanced characterization strategies capable of translating evolving interfacial states into quantitative descriptors. Therefore, although BCR and SBL are physically motivated by interfacial considerations and supported by ablation and SHAP analyses, they should still be regarded as proxy descriptors rather than direct microscopic state variables.

Finally, while SHAP analysis provides a transparent statistical interpretation of feature importance, it does not establish direct physical causality. The synergistic contributions of the redox-active inorganic phase and the conductive polymer network inferred here remain correlation-based. Bridging this gap requires coupling data-driven analysis with physically grounded modeling, such as Density Functional Theory or Molecular Dynamics simulations, to validate interfacial mechanisms suggested by statistical trends. Such integration would enable a transition from correlation-guided screening toward physically constrained materials design.

## 4. Conclusions

This work demonstrates the successful development of a machine learning framework to predict the specific capacitance of composite supercapacitors with high accuracy. By integrating a physically motivated descriptor space with gradient boosting algorithms, the model achieved strong predictive performance within the current dataset. The introduction of two binder-related proxy descriptors, BCR and SBL, improved prediction accuracy and interpretability. Ablation results support that these descriptors provide non-redundant information relevant to interfacial regulation in composite electrodes. Furthermore, the use of SHAP analysis has provided insightful and interpretable results, effectively decoupling the intricate relationships between the binder, active material, and the electrochemical performance. The SHAP analysis shows that BCR and SBL are the dominant features influencing the specific capacitance, with BCR playing a key role in regulating the trade-off between insulation and electronic percolation, while SBL governs ionic accessibility. In summary, this study not only presents an accurate machine learning model for composite supercapacitors but also underscores the significance of binder regulation in performance optimization. By integrating statistical learning with physically meaningful descriptors, this proposed framework shows strong predictive performance within the current literature-derived dataset and offers useful insplit sight into binder-related interfacial regulation. Nevertheless, broader validation on larger and more standardized datasets will be necessary to assess its full generalizability across composite supercapacitor systems.

## Figures and Tables

**Table 1:** Calculation formula for model evaluation indicators.

Metrics	Formula
$R^{2}$	$1-\frac{\sum_{i=1}^{n}\;(\hat{y_{i}}-y_{i}{)}^{2}}{\sum_{i=1}^{n}\;(\overset{¯}{y_{i}}-y_{i}{)}^{2}}$
MAPE	$\frac{100\%}{n}\sum_{i=1}^{n}\;\left|\frac{y_{i}-{\hat{y}}_{l}}{y_{i}}\right|$
MSE	$\frac{1}{n}\sum_{i=1}^{n}\;(y_{i}-{\hat{y}}_{l}{)}^{2}$
RMSE	$\sqrt{\frac{1}{n}\sum_{i=1}^{n}\;(y_{i}-{\hat{y}}_{l}{)}^{2}}$

**Table 2:** Evaluation metrics of linear regression models and decision tree-based models.

Model	R^2^	MAPE	RMSE	Test/Train MSE
Linear Regression	0.3694	82.76	534.40	1.78
Ridge Regression	0.3600	81.34	538.38	1.80
Lasso Regression	0.4874	74.17	434.09	2.05
Elastic Net	0.2322	149.88	531.27	1.74
Bayesian Ridge	0.2409	151.60	528.27	1.71
Decision Tree	0.6695	40.48	386.86	3.82
Random Forest	0.8283	37.94	239.01	3.05
Bagging Regressor	0.9261	35.90	200.02	3.17
ExtraTrees Regression	0.7429	48.51	307.45	3.51

**Table 3:** Evaluation metrics of gradient boosting algorithm and other common models.

Model	R^2^	MAPE	RMSE	Test/Train MSE
AdaBoost	0.8847	59.55	205.91	2.11
GBDT	0.7697	39.85	322.96	5.95
XGBoost	0.9323	26.05	157.78	3.16
LightGBM	0.9218	31.29	169.58	3.46
CatBoost	0.8139	34.40	290.28	9.06
Gaussian Regressor	0.7891	31.25	278.45	4.98
ANN	0.6273	48.87	415.19	3.30
Stacking	0.8155	32.23	289.05	4.97

**Table 4:** Ablation results of five top-performing models without BCR and SBL.

Model	R^2^	MAPE	RMSE	Test/Train MSE
AdaBoost	0.8145	66.84	240.52	2.23
XGBoost	0.8664	32.56	185.73	3.65
LightGBM	0.8411	42.31	212.58	4.14
Bagging Regressor	0.8712	41.66	211.45	3.76
Random Forest	0.7688	48.46	279.25	3.57

**Table 5:** Comparison of the XGBoost model and the Bayesian-optimized XGBoost model.

Model	R^2^	MAPE	RMSE	Test/Train MSE
XGBoost	0.9323	26.05	157.78	3.16
Bayesian-optimized XGBoost	0.9812	14.49	96.46	2.14

## Data Availability

The original contributions presented in this study are included in the article/Supplementary Materials. Further inquiries can be directed to the corresponding authors.

## Supplementary Materials

The following supporting information can be downloaded at: https://www.mdpi.com/article/10.3390/nano16080478/s1, Figure S1: Convergence of the minimum target value with increasing calculations in Bayesian optimization. Table S1: Composite Supercapacitor Dataset. Table S2: Novel Composite Supercapacitor Dataset. Table S3: Predicted Capacitance Values for Novel Composite Supercapacitor Materials Using Various Regression Models. Table S4: Repeated cross-validation evaluation metrics of top-performing models. Table S5: Hyperparameter table for Bayesian-optimized XGBoost model. Table S6: Hyperparameter table for the main baseline models used for comparison. Refs. [46,47,48,49,50,51,52,53,54,55,56,57,58,59,60,61,62,63,64,65,66,67,68,69,70,71,72,73,74,75,76,77,78,79,80,81,82,83,84,85] are cited in the Supplementary Materials.
