# Supplementary material for: Prediction of Composite Supercapacitor Performance Through Combining Machine Learning with Novel Binder-Related Features
Source: Nanomaterials (Basel). 2026 Apr 17;16(8):478. doi: 10.3390/nano16080478 (PMC13119411; doi:10.3390/nano16080478)
Supplement: Supplementary file 1 [file nanomaterials-16-00478-s001.zip › nanomaterials-4246354-supplementary.pdf]

Prediction of Composite Supercapacitor Performance Through Combining Machine Learning with Novel Binder-Related Features

Tianshun Gong [1], Weiyang Yu [2,*] and Xiangfu Wang [1,*]

[1] College of Electronic and Optical Engineering & College of Flexible Electronics (Future Technology), Nanjing University of Posts and Telecommunications, Nanjing 210023, China
[2] School of Physics and Electronic Information, Henan Polytechnic University, Jiaozuo 454003, China
* Correspondence: yuweiyang@hpu.edu.cn (W.Y.); xfwang@njupt.edu.cn (X.W.)

Brief introduction to the mathematical principles of algorithms

1. Linear Regression

Linear regression is one of the foundational algorithms in machine learning, used to model the linear relationship between a dependent variable and independent variables. The classical form of linear regression is represented as:

$$y = \beta_0 + \beta_1 x_1 + \beta_2 x_2 + \cdots + \beta_n x_n + \epsilon \tag{S1}$$

Here, $\boldsymbol{y}$ denotes the target variable, $\boldsymbol{\beta_0}, \boldsymbol{\beta_1}, \dots, \boldsymbol{\beta_n}$ are the regression coefficients, $\boldsymbol{x_1}, \boldsymbol{x_2}, \dots, \boldsymbol{x_n}$ are the independent variables, and $\boldsymbol{\epsilon}$ is the error term. The goal of linear regression is to fit the model by minimizing the sum of squared errors.

2. Ridge Regression

Ridge regression is a linear regression method with L2 regularization. Unlike standard linear regression, ridge regression adds a regularization term to the loss function to prevent overfitting. The objective function is expressed as:

$$\text{Loss} = \sum_{i=1}^{m} (y_i - \hat{y}_i)^2 + \lambda \sum_{j=1}^{n} \beta_j^2 \tag{S2}$$

In this equation, $\boldsymbol{\lambda}$ is the regularization parameter that controls the strength of the penalty term. By introducing L2 regularization, ridge regression effectively constrains the model parameters, reducing the risk of high variance.

3. Lasso Regression

Lasso regression incorporates L1 regularization into the linear regression framework. In Lasso regression, the loss function includes not only the sum of squared residuals but also the sum of the absolute values of the regression coefficients as a penalty term. The formula is as follows:

$$\text{Loss} = \sum_{i=1}^{m} (y_i - \hat{y}_i)^2 + \lambda \sum_{j=1}^{n} \mid \beta_j \mid \tag{S3}$$

A key feature of Lasso regression is its ability to produce sparse solutions, meaning that some coefficients may be shrunk to zero. This property makes Lasso useful for feature selection.

4. Elastic Net

Elastic Net combines both L1 and L2 regularization, drawing on the advantages of both ridge and Lasso regressions. Its loss function is defined as:

$$\text{Loss} = \sum_{i=1}^{m} (y_i - \hat{y}_i)^2 + \lambda_1 \sum_{j=1}^{n} \mid \beta_j \mid + \lambda_2 \sum_{j=1}^{n} \beta_j^2 \tag{S4}$$

Here, $\boldsymbol{\lambda_1}$and $\boldsymbol{\lambda_2}$control the strengths of the L1 and L2 penalties, respectively. Elastic Net is more stable than Lasso when dealing with highly correlated features, and it tends to retain more features in the model.

5. Bayesian Ridge Regression

Bayesian ridge regression is based on the Bayesian inference framework, where the coefficients of ridge regression are treated as random variables. The regression coefficients are estimated by considering the prior distribution and likelihood function. The objective function is expressed as:

$$p(\beta \mid X, y) \propto p(y \mid X, \beta) p(\beta) \tag{S5}$$

In Bayesian ridge regression, the model estimates the coefficients by maximizing the posterior probability, which naturally incorporates the uncertainty in the data.

6. Decision Tree

The Decision Tree algorithm is a fundamental algorithm that uses a tree-like structure to make decisions based on feature values. The objective is to divide the feature space into smaller regions by selecting the best feature at each node. The model is trained using a recursive binary partitioning strategy. The decision tree splits the data into subsets that maximize homogeneity in the target variable at each leaf node. The quality of the splits is typically measured by metrics like Gini impurity or entropy for classification, or mean squared error for regression. The formula for splitting at a node is given as:

$$\text{Gain} = \text{Entropy(Parent)} - \sum(\text{Weighted Entropy(Child)}) \tag{S6}$$

The algorithm continues to split the data until a stopping criterion, such as a maximum tree depth or a minimum number of samples in a node, is reached.

7. Random Forest

Random Forest is an ensemble learning method based on decision trees. It constructs multiple decision trees using bootstrapped samples of the data and then aggregates their predictions. This approach improves model accuracy and stability by reducing the variance typically observed in individual decision trees. The random forest algorithm introduces an additional layer of randomness by selecting a random subset of features for each tree split. The final prediction is obtained by averaging the predictions of all trees in the ensemble. The formula for the Random Forest prediction is:

$$\hat{y} = \frac{1}{B}\sum_{b=1}^{B} \widehat{y_b} \tag{S7}$$

Where $\boldsymbol{B}$ is the total number of decision trees in the forest, and $\widehat{\boldsymbol{y_b}}$ is the prediction of the b-th tree for a given input $\boldsymbol{x}$. This method helps prevent overfitting, especially when dealing with high-dimensional data.

8. Extra Trees Regressor

Extra Trees (Extremely Randomized Trees) is another ensemble learning method that

builds a forest of decision trees. Similar to Random Forest, Extra Trees uses bootstrap sampling, but with a key difference: it makes splits at each node by selecting a random subset of features and choosing the best split randomly, rather than by optimizing a splitting criterion like Gini impurity or mean squared error. This results in trees that are more random and less correlated, which leads to greater model diversity. The final prediction is obtained by averaging the outputs of all the trees, similar to Random Forest. The advantage of Extra Trees over Random Forest is its faster computation, as it does not require optimization at each node split.

9. Bagging Regressor

In Bagging Regressor, the final prediction is typically made by averaging the predictions from all individual models. However, in some cases, the predictions can be weighted based on the performance of each model, where better-performing models have higher weights. The formula for a weighted Bagging Regressor is:

$$\hat{y} = \frac{\sum_{b=1}^{B} w_b \cdot \widehat{y_b}}{\sum_{b=1}^{B} w_b} \tag{S8}$$

Where $\boldsymbol{w_b}$ is the weight associated with the b-th model, often determined by its accuracy or performance, $\boldsymbol{B}$ is the number of base learners (models) in the ensemble, and $\widehat{\boldsymbol{y_b}}$ is the prediction of the b-th model.

10. AdaBoost

AdaBoost, one of the earliest and most widely used boosting algorithms, works by adjusting the weights of the training samples. In each iteration, AdaBoost builds a new model that focuses on the misclassified samples from the previous model. The algorithm assigns higher weights to the misclassified samples, forcing the next model to pay more attention to these harder-to-classify examples. The final prediction is made by taking a weighted vote of the predictions of all the models. The formula for AdaBoost prediction is:

$$\hat{y} = \sum_{m=1}^{M} \alpha_m h_m(x) \tag{S9}$$

Where $\boldsymbol{M}$ is the number of weak learners (iterations), $\boldsymbol{\alpha_m}$ is the weight assigned to the m-th model, typically based on its accuracy, and $\boldsymbol{h_m(x)}$ is the prediction of the m-th model.

AdaBoost is effective in improving the performance of weak learners, especially when they have low variance.

11. GBDT

Gradient Boosting Decision Tree (GBDT) is a boosting algorithm that uses gradient descent to minimize a loss function. In each iteration, GBDT fits a new decision tree to the residuals (the differences between the actual values and the predictions from the previous iteration). GBDT uses decision trees as base learners and optimizes the model by iteratively reducing the residual errors. The objective function for GBDT is:

$$\hat{y} = \sum_{m=1}^{M} \gamma_m h_m(x) \tag{S10}$$

Where $\boldsymbol{M}$ is the number of iterations (decision trees),$\boldsymbol{\gamma_m}$ is the weight for the m-th tree, determined by gradient descent,and $\boldsymbol{h_m(x)}$ is the m-th decision tree.

GBDT is widely used for both regression and classification tasks and performs well even with complex datasets.

12. XGBoost

XGBoost is an optimized version of GBDT that improves both the performance and efficiency of gradient boosting algorithms. It introduces regularization terms in the objective function to prevent overfitting, and it also supports parallelization, making it faster than traditional GBDT. The objective function of XGBoost combines the loss function with a regularization term as follows:

$$\mathcal{L}(\theta) = \sum_{i=1}^{n} L(y_i, \hat{y_i}) + \Omega(\theta) \tag{S11}$$

Where $\boldsymbol{L(y_i, \hat{y}_i)}$ is the loss function (e.g., mean squared error for regression), $\boldsymbol{\Omega(\theta)}$ is the regularization term to control model complexity, typically defined as:

$$\Omega(\theta) = \gamma T + \frac{1}{2}\lambda \sum_{j=1}^{T} w_j^2 \tag{S12}$$

Here, $\boldsymbol{\gamma}$ and $\boldsymbol{\lambda}$ are regularization parameters, $\boldsymbol{T}$ is the number of leaves in the tree, and $\boldsymbol{w_j}$ are the weights of the leaves. The inclusion of regularization helps improve generalization by preventing overfitting.

13.LightGBM

LightGBM is an efficient gradient boosting algorithm designed to be faster and more scalable than XGBoost. It achieves this by using a histogram-based approach to bin continuous features, reducing memory usage and speeding up computation. LightGBM also supports parallel and GPU learning. Its objective function is similar to XGBoost, but LightGBM uses a different tree-growing algorithm, which speeds up the training process. The key advantage of LightGBM is its ability to handle large datasets efficiently while maintaining predictive accuracy. The formula for LightGBM prediction is:

$$\hat{y} = \sum_{m=1}^{M} \gamma_m h_m(x) \tag{S13}$$

Where $\boldsymbol{M}$ is the number of boosting iterations, $\boldsymbol{\gamma_m}$ is the weight for the m-th decision tree, and $\boldsymbol{h_m(x)}$ is the m-th decision tree.

LightGBM is well-suited for large datasets and provides state-of-the-art performance in many Kaggle competitions.

14. CatBoost

CatBoost is a gradient boosting algorithm specifically designed to handle categorical features more efficiently. Unlike traditional gradient boosting algorithms, which require

preprocessing of categorical variables (e.g., one-hot encoding), CatBoost automatically handles categorical features by using an efficient encoding technique that preserves their relationships with the target variable. The core objective function of CatBoost is:

$$\hat{y} = \sum_{m=1}^{M} \gamma_m h_m(x) \tag{S14}$$

Where $\boldsymbol{M}$ is the number of boosting iterations, $\boldsymbol{\gamma_m}$ is the weight for the m-th decision tree,and $\boldsymbol{h_m(x)}$ is the m-th decision tree.

CatBoost's advantage lies in its efficient handling of categorical variables, making it particularly effective in scenarios where categorical data is abundant.

15. Gaussian Process Regressor

The Gaussian Process Regressor (GPR) is a non-parametric, probabilistic regression method that models the relationship between inputs and outputs as a distribution over functions. GPR assumes that the data is generated from a Gaussian process, which defines a distribution over functions. The core advantage of GPR is its ability to provide not only a prediction but also the uncertainty associated with that prediction. The predictive distribution for a new input $\boldsymbol{x_*}$is given by:

$$p(y_* \mid X, y, x_*) = \mathcal{N}(y_* \mid \mu_*, \sigma_*^2) \tag{S15}$$

Where $\boldsymbol{\mu_*}$ is the mean of the posterior distribution, which represents the predicted output,$\boldsymbol{\sigma_*^2}$ is the variance of the posterior distribution, representing the uncertainty of the prediction,$\boldsymbol{X}$ and $\boldsymbol{y}$are the training inputs and outputs, respectively.

GPR is particularly effective when modeling data with noisy observations and when quantifying uncertainty is crucial, such as in applications where predictive confidence is important.

16.ANN (Artificial Neural Network)

Artificial Neural Networks (ANNs) are a class of machine learning models inspired by

the structure and function of the human brain. ANNs consist of layers of interconnected neurons that process input data in a non-linear fashion. The network learns by adjusting the weights of these connections through a process known as backpropagation, using gradient descent to minimize the error between the predicted and actual outputs. The architecture of an ANN can vary, but the most basic form consists of an input layer, one or more hidden layers, and an output layer. The activation function, such as ReLU or sigmoid, introduces non-linearity, enabling the network to learn complex patterns. The general form of an artificial neural network's output is:

$$\hat{y} = f(WX + b) \tag{S16}$$

Where $\boldsymbol{X}$ represents the input features, $\boldsymbol{W}$ is the weight matrix, $\boldsymbol{b}$ is the bias term, and $\boldsymbol{f}$ is the activation function (e.g., ReLU, Sigmoid).

ANNs are powerful tools for modeling complex, non-linear relationships, and they have been successfully applied in a wide range of tasks, including image recognition, natural language processing, and time series forecasting.

17. Stacking (Stacked Generalization)

Stacking is an ensemble learning strategy that combines multiple base learners to improve the performance of a model. Unlike bagging or boosting, where the base models are trained independently or sequentially, stacking trains multiple base models and then uses a meta-model to combine their predictions. The base models can be of different types (e.g., decision trees, linear models, neural networks), and the meta-model typically learns how to best combine the outputs of the base models. The final prediction is made by the meta-model, which is trained on the predictions from the base learners. The formula for stacking prediction is:

$$\hat{y} = g(\widehat{y_1}, \widehat{y_2}, \ldots, \widehat{y_n}) \tag{S17}$$

Where $\widehat{\boldsymbol{y_1}}, \widehat{\boldsymbol{y_2}}, \ldots, \widehat{\boldsymbol{y_n}}$ are the predictions from the base learners, $\boldsymbol{g}$ is the meta-model that combines the predictions, typically a regression model or another classifier.

Table S1. Composite Supercapacitor Dataset.

Name	SSA (m²/g)	Lattice _A(Å)	Strain (%)	Electrolyte	Binder Type	Binder _wt (%)	Cond _wt (%)	BCR	SBL (mg/m²)	Solvent	T_dry (°C)	t_dry (h)	J_load (A/g)	V_win (V)	Pore Type	Specific Capacitance (F /g)	Ref.
T-Nb_2O_5	100.1	6.3	1.3	0	PTFE	5	15	0.333	0.500	Ethanol	110	12	1	2.5	1	366	[46]
T-Nb_2O_5	100.1	6.3	1.3	0	PTFE	5	15	0.333	0.500	Ethanol	110	12	2	2.5	1	354	[46]
T-Nb_2O_5	100.1	6.3	1.3	0	PTFE	5	15	0.333	0.500	Ethanol	110	12	5	2.5	1	338	[46]
T-Nb_2O_5	100.1	6.3	1.3	0	PTFE	5	15	0.333	0.500	Ethanol	110	12	10	2.5	1	246	[46]
MoS_2	21.1	3.16	2.44	0	PTFE	10	15	0.667	4.739	Ethanol	80	6	1	1	0	129.2	[47]
MoS_2	21.1	3.16	2.44	0	PTFE	10	15	0.667	4.739	Ethanol	80	6	1.5	1	0	115.294	[47]
MoS_2	21.1	3.16	2.44	0	PTFE	10	15	0.667	4.739	Ethanol	80	6	3	1	0	102.353	[47]
MoS_2	21.1	3.16	2.44	0	PTFE	10	15	0.667	4.739	Ethanol	80	6	10	1	0	73.8	[47]
MoOx@NiS_2	91.9	5.6	6.06	1	PVDF	10	10	1	1.088	NMP	60	12	1	0.6	0	1050	[48]
MoOx@NiS_2	91.9	5.6	6.06	1	PVDF	10	10	1	1.088	NMP	60	12	2	0.6	0	946	[48]
MoOx@NiS_2	91.9	5.6	6.06	1	PVDF	10	10	1	1.088	NMP	60	12	5	0.6	0	833	[48]
MoOx@NiS_2	91.9	5.6	6.06	1	PVDF	10	10	1	1.088	NMP	60	12	10	0.6	0	708	[48]
FeOOH-CNT-II	96.225	8.56	0.69	1	PVDF	10	20	0.5	1.039	NMP	80	10	1	0.4	0	1550	[49]
FeOOH-CNT-II	96.225	8.56	0.69	1	PVDF	10	20	0.5	1.039	NMP	80	10	2	0.4	0	1550	[49]
FeOOH-CNT-II	96.225	8.56	0.69	1	PVDF	10	20	0.5	1.039	NMP	80	10	5	0.4	0	1500	[49]
FeOOH-CNT-II	96.225	8.56	0.69	1	PVDF	10	20	0.5	1.039	NMP	80	10	10	0.4	0	1450	[49]
FeOOH-CNT-II	96.225	8.56	0.69	1	PVDF	10	20	0.5	1.039	NMP	80	10	20	0.4	0	1380	[49]
$(Ni_{0.5}Co_{0.5})(OH)_2$ /$Co(BO_2)_2$	217.1	3.12	0.19	1	PVDF	5	25	0.2	0.230	Ethanol	50	8	1	0.6	0	2257	[50]
$(Ni_{0.5}Co_{0.5})(OH)_2$ /$Co(BO_2)_2$	217.1	3.12	0.19	1	PVDF	5	25	0.2	0.230	Ethanol	50	8	3	0.6	0	2186	[50]
$(Ni_{0.5}Co_{0.5})(OH)_2$ /$Co(BO_2)_2$	217.1	3.12	0.19	1	PVDF	5	25	0.2	0.230	Ethanol	50	8	5	0.6	0	2157	[50]
$(Ni_{0.5}Co_{0.5})(OH)_2$ /$Co(BO_2)_2$	217.1	3.12	0.19	1	PVDF	5	25	0.2	0.230	Ethanol	50	8	10	0.6	0	2108	[50]
$(Ni_{0.5}Co_{0.5})(OH)_2$ /$Co(BO_2)_2$	217.1	3.12	0.19	1	PVDF	5	25	0.2	0.230	Ethanol	50	8	20	0.6	0	2071	[50]
CNTF@$Co(OH)_2$	391.4	6.05	0.5	1	PTFE	5	10	0.5	0.128	Ethanol	120	6	0.5	0.5	1	614.3	[51]
CNTF@$Co(OH)_2$	391.4	6.05	0.5	1	PTFE	5	10	0.5	0.128	Ethanol	120	6	1	0.5	1	551.9	[51]

CNTF@Co(OH)$_2$	391.4	6.05	0.5	1	PTFE	5	10	0.5	0.128	Ethanol	120	6	3	0.5	1	528	[51]
CNTF@Co(OH)$_2$	391.4	6.05	0.5	1	PTFE	5	10	0.5	0.128	Ethanol	120	6	5	0.5	1	466.2	[51]
CNTF@Co(OH)$_2$	391.4	6.05	0.5	1	PTFE	5	10	0.5	0.128	Ethanol	120	6	10	0.5	1	444.5	[51]
CNTF@Co(OH)$_2$	391.4	6.05	0.5	1	PTFE	5	10	0.5	0.128	Ethanol	120	6	10	0.5	1	444.5	[51]
H-NNMO	75.5	4.14	0.89	1	PVDF	10	50	0.2	1.325	NMP	100	12	1	0.4	1	677.8	[52]
M-NNMO	147.9	4.12	1.36	1	PVDF	10	50	0.2	0.676	NMP	100	12	1	0.4	1	1147.5	[52]
Mn_2O_3/C	22.491	9.41	4.1	0	PTFE	5	15	0.333	2.223	Ethanol	150	6	1	1.2	0	277	[53]
Mn_2O_3/C	22.491	9.41	4.1	0	PTFE	5	15	0.333	2.223	Ethanol	150	6	2	1.2	0	242.67	[53]
Mn_2O_3/C	22.491	9.41	4.1	0	PTFE	5	15	0.333	2.223	Ethanol	150	6	4	1.2	0	188.67	[53]
Mn_2O_3/C	22.491	9.41	4.1	0	PTFE	5	15	0.333	2.223	Ethanol	150	6	5	1.2	0	157.5	[53]
Mn_2O_3/C	22.491	9.41	4.1	0	PTFE	5	15	0.333	2.223	Ethanol	150	6	6	1.2	0	130	[53]
NiO@2%Ce	4.3451	4.298	3.12	1	PVDF	10	10	1	23.014	NMP	80	8	1	0.7	0	122	[54]
NiO@2%Ce	4.3451	4.298	3.12	1	PVDF	10	10	1	23.014	NMP	80	8	2	0.7	0	108	[54]
NiO@2%Ce	4.3451	4.298	3.12	1	PVDF	10	10	1	23.014	NMP	80	8	3	0.7	0	85	[54]
NiO@2%Ce	4.3451	4.298	3.12	1	PVDF	10	10	1	23.014	NMP	80	8	4	0.7	0	43	[54]
NiO@2%Ce	4.3451	4.298	3.12	1	PVDF	10	10	1	23.014	NMP	80	8	5	0.7	0	32	[54]
CeO_2/NC-32–450	618.85	5.3	2.03	1	PVDF	10	0	1	0.162	NMP	120	12	0.5	1	0	275.3	[55]
MnNi-5	24	5.66	1.74	1	PVDF	10	10	1	4.167	NMP	120	12	1	0.5	0	707	[56]
MnNi-5	24	5.66	1.74	1	PVDF	10	10	1	4.167	NMP	120	12	2	0.5	0	687	[56]
MnNi-5	24	5.66	1.74	1	PVDF	10	10	1	4.167	NMP	120	12	4	0.5	0	685	[56]
MnNi-5	24	5.66	1.74	1	PVDF	10	10	1	4.167	NMP	120	12	8	0.5	0	596	[56]
MnNi-5	24	5.66	1.74	1	PVDF	10	10	1	4.167	NMP	120	12	12	0.5	0	457	[56]
MnNi-5	24	5.66	1.74	1	PVDF	10	10	1	4.167	NMP	120	12	16	0.5	0	342	[56]
Mn_3O_4-CeO_2Holey-graphene	160.21	5.79	2.5	0	PVDF	10	10	1	0.624	NMP	120	8	2	2	0	310	[57]
V_2CT_x/NiV-LDH	74.992	3.08	1.28	1	PTFE	10	10	1	1.33	Ethanol	60	8	1	1.5	0	1658.19	[58]
V_2CT_x/NiV-LDH	74.992	3.08	1.28	1	PTFE	10	10	1	1.333	Ethanol	60	8	2	1.5	0	1463.6	[58]
V_2CT_x/NiV-LDH	74.992	3.08	1.28	1	PTFE	10	10	1	1.333	Ethanol	60	8	3	1.5	0	1378.2	[58]
V_2CT_x/NiV-LDH	74.992	3.08	1.28	1	PTFE	10	10	1	1.333	Ethanol	60	8	5	1.5	0	1170	[58]
V_2CT_x/NiV-LDH	74.992	3.08	1.28	1	PTFE	10	10	1	1.333	Ethanol	60	8	8	1.5	0	852.795	[58]
V_2CT_x/NiV-LDH	74.992	3.08	1.28	1	PTFE	10	10	1	1.333	Ethanol	60	8	10	1.5	0	680	[58]
NC_2/NCNTs	82.33	7.87	3	1	PVA	10	10	1	1.214	Water	100	6	2	0.5	0	73	[59]

NC_2/NCNTs	82.33	7.87	3	1	PVA	10	10	1	1.215	Water	100	6	4	0.5	0	67	[59]
NC_2/NCNTs	82.33	7.87	3	1	PVA	10	10	1	1.215	Water	100	6	6	0.5	0	62	[59]
NC_2/NCNTs	82.33	7.87	3	1	PVA	10	10	1	1.215	Water	100	6	8	0.5	0	58	[59]
NC_2/NCNTs	82.33	7.87	3	1	PVA	10	10	1	1.215	Water	100	6	10	0.5	0	47	[59]
NC_2/NCNTs	82.33	7.87	3	1	PVA	10	10	1	1.215	Water	100	6	14	0.5	0	43	[59]
V_2O_5/CNTs–SAC	78	7.11	0.5	0	PVDF	20	30	0.667	2.564	Ethanol	80	6	10	0.8	0	357.5	[60]
$SmNiO_3$/SWCNT	11.223	5.08	6.45	1	PVDF	10	20	0.5	8.910	NMP	80	12	1	1.5	0	170.58	[61]
$SmNiO_3$/SWCNT	11.223	5.08	6.45	1	PVDF	10	20	0.5	8.910	NMP	80	12	2	1.5	0	156.45	[61]
$SmNiO_3$/SWCNT	11.223	5.08	6.45	1	PVDF	10	20	0.5	8.910	NMP	80	12	3	1.5	0	141.54	[61]
$SmNiO_3$/SWCNT	11.223	5.08	6.45	1	PVDF	10	20	0.5	8.910	NMP	80	12	4	1.5	0	137.52	[61]
$SmNiO_3$/SWCNT	11.223	5.08	6.45	1	PVDF	10	20	0.5	8.910	NMP	80	12	5	1.5	0	133.96	[61]
$SmNiO_3$/SWCNT	11.223	5.08	6.45	1	PVDF	10	20	0.5	8.910	NMP	80	12	10	1.5	0	106.6	[61]
$SmNiO_3$/SWCNT	11.223	5.08	6.45	1	PVDF	10	20	0.5	8.910	NMP	80	12	15	1.5	0	93.3	[61]
$SmNiO_3$/SWCNT	11.223	5.08	6.45	1	PVDF	10	20	0.5	8.910	NMP	80	12	20	1.5	0	80.93	[61]
Co_3O_4 nanowire	13.6	8.07	0.21	1	PVDF	10	15	0.667	7.353	NMP	60	24	1	0.5	0	1110	[62]
NCMO-SSNRs	34.1	5.08	1.17	1	PTFE	12.5	25	0.5	3.666	Ethanol	60	8	5	0.4	0	665	[63]
NCMO-SSNRs	34.1	5.08	1.17	1	PTFE	12.5	25	0.5	3.666	Ethanol	60	8	1	0.4	0	981	[63]
NCMO-SSNRs	34.1	5.08	1.17	1	PTFE	12.5	25	0.5	3.666	Ethanol	60	8	2	0.4	0	896.3	[63]
NCMO-SSNRs	34.1	5.08	1.17	1	PTFE	12.5	25	0.5	3.666	Ethanol	60	8	3	0.4	0	791.4	[63]
NCMO-SSNRs	34.1	5.08	1.17	1	PTFE	12.5	25	0.5	3.666	Ethanol	60	8	4	0.4	0	714.5	[63]
Mo_2C	11	3.12	0.97	1	PTFE	10	10	1	9.091	Ethanol	60	1	0.1	1	0	206	[64]
PAC/MnO_2	643	4.37	0.59	0	PVDF	10	10	1	0.156	NMP	60	8	0.5	1	1	208.75	[65]
Ni-Cu(I)	256.76	9.58	0.41	1	PVDF	10	10	1	0.389	Ethanol	60	12	1	1.8	0	1226.5	[66]
Ni-Cu(I)	256.76	9.58	0.41	1	PVDF	10	10	1	0.389	Ethanol	60	12	2	1.8	0	1102.4	[66]
Ni-Cu(I)	256.76	9.58	0.41	1	PVDF	10	10	1	0.389	Ethanol	60	12	3	1.8	0	1006.1	[66]
Ni-Cu(I)	256.76	9.58	0.41	1	PVDF	10	10	1	0.389	Ethanol	60	12	4	1.8	0	897.9	[66]
Ni-Cu(I)	256.76	9.58	0.41	1	PVDF	10	10	1	0.389	Ethanol	60	12	5	1.8	0	868.2	[66]
BaS/N-rGO	82.21	6.1	2.01	1	PVDF	10	10	1	1.216	Ethanol	60	12	1	1	0	913.3	[67]
ZnS NS	120	5.35	1.11	1	PTFE	6	14	0.428	0.5	Ethanol	90	9	1	1	0	2282	[68]
ZnS NS	120	5.35	1.11	1	PTFE	6	14	0.428	0.5	Ethanol	90	9	2	1	0	1572	[68]
ZnS NS	120	5.35	1.11	1	PTFE	6	14	0.428	0.5	Ethanol	90	9	4	1	0	1312	[68]
ZnS NS	120	5.35	1.11	1	PTFE	6	14	0.428	0.5	Ethanol	90	9	8	1	0	1216	[68]

ZnS NS	120	5.35	1.11	1	PTFE	6	14	0.428	0.5	Ethanol	90	9	12	1	0	1176	[68]
CeO_2-Co_3O_4	28.58	8.07	0.12	1	PVDF	10	10	1	3.499	NMP	60	12	1	0.5	0	603.3	[69]
NiCo/NMCS-50	660.9	3.55	0.28	1	PVDF	10	10	1	0.151	NMP	90	8	1	2	0	585	[70]
5Sn-Co_3O_4	143	8.13	0.62	1	PTFE	10	10	1	0.699	Ethanol	70	10	1	0.8	0	900	[71]
S-NCS-4	3357	3.6	46.3	1	PTFE	10	10	1	0.030	Ethanol	105	12	0.5	1	1	405	[72]
S-NCS-4	3357	3.6	46.3	1	PTFE	10	10	1	0.030	Ethanol	105	12	1	1	1	357	[72]
S-NCS-4	3357	3.6	46.3	1	PTFE	10	10	1	0.030	Ethanol	105	12	2	1	1	317	[72]
S-NCS-4	3357	3.6	46.3	1	PTFE	10	10	1	0.030	Ethanol	105	12	5	1	1	294	[72]
S-NCS-4	3357	3.6	46.3	1	PTFE	10	10	1	0.030	Ethanol	105	12	10	1	1	286	[72]
S-NCS-4	3357	3.6	46.3	1	PTFE	10	10	1	0.030	Ethanol	105	12	20	1	1	276	[72]
S-NCS-4	3357	3.6	46.3	1	PTFE	10	10	1	0.030	Ethanol	105	12	40	1	1	268	[72]
10-rGO/$NimoO_4$	39.142	9.58	0.42	1	PVDF	5	10	0.5	1.277	NMP	80	12	3	0.5	0	680	[73]
10-rGO/$NimoO_4$	39.142	9.58	0.42	1	PVDF	5	10	0.5	1.277	NMP	80	12	4	0.5	0	616	[73]
10-rGO/$NimoO_4$	39.142	9.58	0.42	1	PVDF	5	10	0.5	1.277	NMP	80	12	5	0.5	0	605	[73]
10-rGO/$NimoO_4$	39.142	9.58	0.42	1	PVDF	5	10	0.5	1.277	NMP	80	12	6	0.5	0	593	[73]
10-rGO/$NimoO_4$	39.142	9.58	0.42	1	PVDF	5	10	0.5	1.277	NMP	80	12	7	0.5	0	575	[73]
5-rGO/$NimoO_4$	34.903	9.58	0.42	1	PVDF	5	10	0.5	1.433	NMP	80	12	2	0.5	0	380	[73]
5-rGO/$NimoO_4$	34.903	9.58	0.42	1	PVDF	5	10	0.5	1.433	NMP	80	12	3	0.5	0	350	[73]
5-rGO/$NimoO_4$	34.903	9.58	0.42	1	PVDF	5	10	0.5	1.433	NMP	80	12	4	0.5	0	331	[73]
5-rGO/$NimoO_4$	34.903	9.58	0.42	1	PVDF	5	10	0.5	1.433	NMP	80	12	5	0.5	0	311	[73]
5-rGO/$NimoO_4$	34.903	9.58	0.42	1	PVDF	5	10	0.5	1.433	NMP	80	12	6	0.5	0	296	[73]
15-rGO/$NimoO_4$	10.346	9.58	0.42	1	PVDF	5	10	0.5	4.833	NMP	80	12	2	0.5	0	582	[73]
15-rGO/$NimoO_4$	10.346	9.58	0.42	1	PVDF	5	10	0.5	4.833	NMP	80	12	3	0.5	0	574	[73]
15-rGO/$NimoO_4$	10.346	9.58	0.42	1	PVDF	5	10	0.5	4.833	NMP	80	12	4	0.5	0	550	[73]
15-rGO/$NimoO_4$	10.346	9.58	0.42	1	PVDF	5	10	0.5	4.833	NMP	80	12	5	0.5	0	533	[73]
15-rGO/$NimoO_4$	10.346	9.58	0.42	1	PVDF	5	10	0.5	4.833	NMP	80	12	6	0.5	0	520	[73]
15-rGO/$NimoO_4$	10.346	9.58	0.42	1	PVDF	5	10	0.5	4.833	NMP	80	12	7	0.5	0	513	[73]
$Ni_3Si_2O_5(OH)_4$	67.6	5.22	2.25	1	PVDF	10	10	1	1.479	NMP	60	12	1	0.8	1	178.9	[74]
$Ni_3Si_2O_5(OH)_4$	67.6	5.22	2.25	1	PVDF	10	10	1	1.479	NMP	60	12	2	0.8	1	166.5	[74]
$Ni_3Si_2O_5(OH)_4$	67.6	5.22	2.25	1	PVDF	10	10	1	1.479	NMP	60	12	3	0.8	1	150.8	[74]
$Ni_3Si_2O_5(OH)_4$	67.6	5.22	2.25	1	PVDF	10	10	1	1.479	NMP	60	12	4	0.8	1	138.9	[74]
$Ni_3Si_2O_5(OH)_4$	67.6	5.22	2.25	1	PVDF	10	10	1	1.479	NMP	60	12	5	0.8	1	132.5	[74]

$Ni_3Si_2O_5(OH)_4$	67.6	5.22	2.25	1	PVDF	10	10	1	1.479	NMP	60	12	6	0.8	1	126.8	[74]
$Ni_3Si_2O_5(OH)_4$	67.6	5.22	2.25	1	PVDF	10	10	1	1.479	NMP	60	12	8	0.8	1	120.1	[74]
GP-P0	32.8	5.78	0.34	0	PTFE	5	10	0.5	1.524	Ethanol	60	8	0.5	1	0	118.5	[75]
GP-P1	34.5	5.78	0.34	0	PTFE	5	10	0.5	1.449	Ethanol	60	8	0.5	1	0	177.2	[75]
GP-P3	37.6	5.78	0.34	0	PTFE	5	10	0.5	1.330	Ethanol	60	8	0.5	1	0	215.8	[75]
GP-P5	36.8	5.78	0.34	0	PTFE	5	10	0.5	1.359	Ethanol	60	8	0.5	1	0	158.2	[75]
GP-P0	32.8	5.78	0.34	0	PTFE	5	10	0.5	1.524	Ethanol	60	8	0.8	1	0	86.9	[75]
GP-P1	34.5	5.78	0.34	0	PTFE	5	10	0.5	1.449	Ethanol	60	8	0.8	1	0	152	[75]
GP-P3	37.6	5.78	0.34	0	PTFE	5	10	0.5	1.330	Ethanol	60	8	0.8	1	0	205.8	[75]
GP-P5	36.8	5.78	0.34	0	PTFE	5	10	0.5	1.359	Ethanol	60	8	0.8	1	0	130.1	[75]
GP-P0	32.8	5.78	0.34	0	PTFE	5	10	0.5	1.524	Ethanol	60	8	1	1	0	71.6	[75]
GP-P1	34.5	5.78	0.34	0	PTFE	5	10	0.5	1.449	Ethanol	60	8	1	1	0	143.5	[75]
GP-P3	37.6	5.78	0.34	0	PTFE	5	10	0.5	1.330	Ethanol	60	8	1	1	0	201.5	[75]
GP-P5	36.8	5.78	0.34	0	PTFE	5	10	0.5	1.359	Ethanol	60	8	1	1	0	113.9	[75]
GP-P0	32.8	5.78	0.34	0	PTFE	5	10	0.5	1.524	Ethanol	60	8	1.5	1	0	55.1	[75]
GP-P1	34.5	5.78	0.34	0	PTFE	5	10	0.5	1.449	Ethanol	60	8	1.5	1	0	124.7	[75]
GP-P3	37.6	5.78	0.34	0	PTFE	5	10	0.5	1.330	Ethanol	60	8	1.5	1	0	183.8	[75]
GP-P5	36.8	5.78	0.34	0	PTFE	5	10	0.5	1.359	Ethanol	60	8	1.5	1	0	97.2	[75]
GP-P0	32.8	5.78	0.34	0	PTFE	5	10	0.5	1.524	Ethanol	60	8	2	1	0	44.1	[75]
GP-P1	34.5	5.78	0.34	0	PTFE	5	10	0.5	1.449	Ethanol	60	8	2	1	0	95.6	[75]
GP-P3	37.6	5.78	0.34	0	PTFE	5	10	0.5	1.330	Ethanol	60	8	2	1	0	168.7	[75]
GP-P5	36.8	5.78	0.34	0	PTFE	5	10	0.5	1.359	Ethanol	60	8	2	1	0	82.6	[75]
GP-P0	32.8	5.78	0.34	0	PTFE	5	10	0.5	1.524	Ethanol	60	8	5	1	0	30.2	[75]
GP-P1	34.5	5.78	0.34	0	PTFE	5	10	0.5	1.449	Ethanol	60	8	5	1	0	74.9	[75]
GP-P3	37.6	5.78	0.34	0	PTFE	5	10	0.5	1.330	Ethanol	60	8	5	1	0	157.2	[75]
GP-P5	36.8	5.78	0.34	0	PTFE	5	10	0.5	1.359	Ethanol	60	8	5	1	0	63.1	[75]
Mn_3O_4@N-doped carbon/graphene (mCG)	326	5.8	0.69	0	PTFE	10	10	1	0.307	Ethanol	60	12	1	1	1	456	[76]
Mn_3O_4@N-doped carbon/graphene (mCG)	326	5.8	0.69	0	PTFE	10	10	1	0.307	Ethanol	60	12	2	1	1	398	[76]

Mn_3O_4@N-doped carbon/graphene (mCG)	326	5.8	0.69	0	PTFE	10	10	1	0.307	Ethanol	60	12	5	1	1	348	[76]
Mn_3O_4@N-doped carbon/graphene (mCG)	326	5.8	0.69	0	PTFE	10	10	1	0.307	Ethanol	60	12	10	1	1	298	[76]
Mn_3O_4@N-doped carbon/graphene (mCG)	326	5.8	0.69	0	PTFE	10	10	1	0.307	Ethanol	60	12	20	1	1	246	[76]
CNCs-700	1088	4.26	0.47	1	PVDF	10	10	1	0.092	NMP	60	12	1	1	1	245	[77]
CNCs-800	1001	4.26	0.47	1	PVDF	10	10	1	0.100	NMP	60	12	1	1	1	248	[77]
CNCs-800	1001	4.26	0.47	1	PVDF	10	10	1	0.100	NMP	60	12	10	1	1	217	[77]
CNCs-800	1001	4.26	0.47	1	PVDF	10	10	1	0.100	NMP	60	12	100	1	1	189	[77]
CNCs-900	769	4.26	0.47	1	PVDF	10	10	1	0.130	NMP	60	12	1	1	1	262	[77]
($Ni(OH)_2$- MnO_2-RGO)	147	3.12	0.43	1	PTFE	5	23	0.217	0.340	Water	80	12	2	0.5	0	1985	[78]
($Ni(OH)_2$- MnO_2-RGO)	147	3.12	0.43	1	PTFE	5	23	0.217	0.340	Water	80	12	3	0.5	0	1736	[78]
($Ni(OH)_2$- MnO_2-RGO)	147	3.12	0.43	1	PTFE	5	23	0.217	0.340	Water	80	12	4	0.5	0	1501	[78]
($Ni(OH)_2$- MnO_2-RGO)	147	3.12	0.43	1	PTFE	5	23	0.217	0.340	Water	80	12	6	0.5	0	1062	[78]
($Ni(OH)_2$- MnO_2-RGO)	147	3.12	0.43	1	PTFE	5	23	0.217	0.340	Water	80	12	8	0.5	0	724	[78]
CA-L0	796	3.791	13.2	1	PVDF	10	10	1	0.126	NMP	80	12	0.5	1	1	107.3	[79]
CA-L0	796	3.791	13.2	1	PVDF	10	10	1	0.126	NMP	80	12	1	1	1	105	[79]
CA-L20	779	3.349	0.03	1	PVDF	10	10	1	0.128	NMP	80	12	0.5	1	1	142.8	[79]
CA-L20	779	3.349	0.03	1	PVDF	10	10	1	0.128	NMP	80	12	1	1	1	140.5	[79]
CA-L40	533	3.166	5.5	1	PVDF	10	10	1	0.188	NMP	80	12	0.5	1	1	126.4	[79]
CA-L40	533	3.166	5.5	1	PVDF	10	10	1	0.188	NMP	80	12	1	1	1	122.5	[79]
CA-L60	512	3.149	5.9	1	PVDF	10	10	1	0.195	NMP	80	12	0.5	1	1	90.4	[79]
CA-L60	512	3.149	5.9	1	PVDF	10	10	1	0.195	NMP	80	12	1	1	1	87.6	[79]
PGBC-1	1732	2.13	0.89	1	PVDF	10	10	1	0.058	NMP	120	8	0.5	1	0	222	[80]
PGBC-1	1732	2.13	0.89	1	PVDF	10	10	1	0.058	NMP	120	8	1	1	0	171.4	[80]
PGBC-1	1732	2.13	0.89	1	PVDF	10	10	1	0.058	NMP	120	8	2	1	0	148.6	[80]

PGBC-1	1732	2.13	0.89	1	PVDF	10	10	1	0.058	NMP	120	8	5	1	0	127	[80]
PGBC-1	1732	2.13	0.89	1	PVDF	10	10	1	0.058	NMP	120	8	8	1	0	121.6	[80]
PGBC-1	1732	2.13	0.89	1	PVDF	10	10	1	0.058	NMP	120	8	10	1	0	118.1	[80]
PGBC-1	1732	2.13	0.89	1	PVDF	10	10	1	0.058	NMP	120	8	20	1	0	115	[80]
PAC-4:1	3550.7	3.62	15.2	1	PTFE	10	10	1	0.028	Ethanol	60	12	1	1	1	373	[81]
PAC-4:1	3550.7	3.62	15.2	1	PTFE	10	10	1	0.028	Ethanol	60	12	2	1	1	335	[81]
PAC-4:1	3550.7	3.62	15.2	1	PTFE	10	10	1	0.028	Ethanol	60	12	4	1	1	314	[81]
PAC-4:1	3550.7	3.62	15.2	1	PTFE	10	10	1	0.028	Ethanol	60	12	6	1	1	308	[81]
PAC-4:1	3550.7	3.62	15.2	1	PTFE	10	10	1	0.028	Ethanol	60	12	10	1	1	293	[81]
PAC-4:1	3550.7	3.62	15.2	1	PTFE	10	10	1	0.028	Ethanol	60	12	15	1	1	279	[81]
PAC-4:1	3550.7	3.62	15.2	1	PTFE	10	10	1	0.028	Ethanol	60	12	20	1	1	272	[81]
PAC-4:1	3550.7	3.62	15.2	1	PTFE	10	10	1	0.028	Ethanol	60	12	40	1	1	244	[81]
PTAC-x	2670.8	3.64	15.2	1	PTFE	10	10	1	0.037	Ethanol	60	12	1	1	1	346.1	[82]
PTAC-x	2670.8	3.64	15.2	1	PTFE	10	10	1	0.037	Ethanol	60	12	3	1	1	290.5	[82]
PTAC-x	2670.8	3.64	15.2	1	PTFE	10	10	1	0.037	Ethanol	60	12	5	1	1	268	[82]
PTAC-x	2670.8	3.64	15.2	1	PTFE	10	10	1	0.037	Ethanol	60	12	7	1	1	261.2	[82]
PTAC-x	2670.8	3.64	15.2	1	PTFE	10	10	1	0.037	Ethanol	60	12	10	1	1	253	[82]
PTAC-x	2670.8	3.64	15.2	1	PTFE	10	10	1	0.037	Ethanol	60	12	20	1	1	242	[82]
PTAC-x	2670.8	3.64	15.2	1	PTFE	10	10	1	0.037	Ethanol	60	12	50	1	1	214.5	[82]

*(Electrolyte: 0 indicates neutral, and 1 indicates alkaline; Pore Type: 0 indicates mesoporous pores, and 1 indicates hierarchical pores.)

Table S2. Novel Composite Supercapacitor Dataset.

Name	SSA (m^2/g)	Lattice _A(Å)	Strain (%)	Electrolyte	Binder Type	Binder _wt (%)	Cond _wt (%)	BCR	SBL (mg/m^2)	Solvent	T_dry (°C)	t_dry (h)	J_load (A/g)	V_win (V)	Pore Type	Specific Capacitance (F /g)	Ref.
$Bi3Y_{0.80}Sm_{0.20}O_6$	252.6	5.5	1.85	1	PDVF	10	10	1	0.396	NMP	50	12	1	0.6	1	1150.24	[83]
PC-40	1147.6	2.06	11.12	1	PTFE	10	10	1	0.087	Ethanol	80	12	1	1	0	225.5	[84]
FL-MoS_2 @rGO-2	26.58	3.11	1.58	0	PVDF	10	10	1	3.762	NMP	60	12	2	0.9	0	160.8	[85]
NiO/ZrO_2	5.23	4.16	0.24	1	PTFE	10	10	1	19.120	Ethanol	80	12	2	1	1	342	[86]

*(Electrolyte: 0 indicates neutral, and 1 indicates alkaline; Pore Type: 0 indicates mesoporous pores, and 1 indicates hierarchical pores.

Table S3. Predicted Capacitance Values for Novel Composite Supercapacitor Materials Using Various Regression Models

Name	AdaBoost (F/g)	Bagging Regressor (F/g)	LightGBM (F/g)	XGBoost (F/g)	Random Forest (F/g)
$Bi3Y_{0.80}Sm_{0.20}O_6$	1304.02	1395.12	1370.21	1150.24	1211.07
PC-40	212.94	199.95	267.73	225.5	192.48
FL-MoS_2 @rGO-2	180.97	129.03	144.11	160.8	172.10
NiO/ZrO_2	292.62	410.02	396.62	358.72	396.62

Table S4. Repeated cross-validation evaluation metrics of top-performing models.

Model	R^2	MAPE	RMSE	Test/Train MSE
XGBoost	0.904 ± 0.027	29.8 ± 4.6	181.4±22.7	2.78 ± 0.46
LightGBM	0.892 ± 0.031	33.4 ± 5.1	194.6 ± 25.9	2.96 ± 0.51
Bagging Regressor	0.887 ± 0.034	36.2 ± 5.8	206.8 ± 28.4	2.84 ± 0.49
Random Forest	0.853 ± 0.041	39.7 ± 6.4	233.5 ± 33.8	2.91 ± 0.57
AdaBoost	0.836 ± 0.049	57.9 ± 8.2	246.1 ± 38.7	2.35 ± 0.44

Table S5. Hyperparameter table for Bayesian-optimized XGBoost model.

Hyperparameter	Value	Role in model	Status
objective	reg:squarederror	Objective function for continuous capacitance prediction	Optimized setup
n_estimators	200	Number of boosting trees	Bayesian optimized
max_depth	5	Maximum tree depth controlling model complexity	Bayesian optimized
learning_rate	0.11899	Shrinkage factor for each boosting step	Bayesian optimized
subsample	0.64586	Row subsampling ratio per tree	Bayesian optimized
colsample_bytree	0.62297	Feature subsampling ratio per tree	Bayesian optimized
min_child_weight	3	Minimum instance weight required in a child node	Bayesian optimized
gamma	1.14600	Minimum loss reduction required for split	Bayesian optimized
reg_alpha	7.39033	L1 regularization term	Bayesian optimized
reg_lambda	7.90319	L2 regularization term	Bayesian optimized
random_state	42	Seed used for stochastic reproducibility	Report explicitly

Table S6. Hyperparameter table for the main baseline models used for comparison.

Model	Core hyperparameters	Value(s)	Reproducibility item	Seed
Random Forest	n_estimators; max_depth; max_features	300; None; sqrt	Train/test split fixed	42
LightGBM	n_estimators; learning_rate; num_leaves	250; 0.05; 31	Training-only tuning	42
AdaBoost	n_estimators; learning_rate; loss	200; 0.05; linear	Training-only tuning	42
Bagging Regressor	n_estimators; max_samples; max_features	200; 0.80; 1.00	Training-only tuning	42
Gradient Boosting	n_estimators; learning_rate; max_depth	200; 0.05; 3	Training-only tuning	42

Bayesian optimization is a probabilistic method used for global optimization, commonly applied in machine learning for hyperparameter tuning. It creates a surrogate model of the objective function, frequently employing Gaussian processes, to estimate the performance of various hyperparameter combinations. An acquisition function is then used to choose the most promising hyperparameters for testing. This process strikes a balance between exploring new possibilities and exploiting areas with known high performance. In contrast to grid search or random search, Bayesian optimization minimizes unnecessary evaluations, making it more computationally efficient and resource-saving.

Figure S1 shows the trends of the observed (blue line) and estimated (green line) minimum target values during optimization. Results are shown after 50 iterations (Figure S1A), 100 iterations (Figure S1B), and 150 iterations (Figure S1C). As the number of iterations increases, the two lines converge. This convergence indicates that the optimization process is progressively finding the optimal solution.

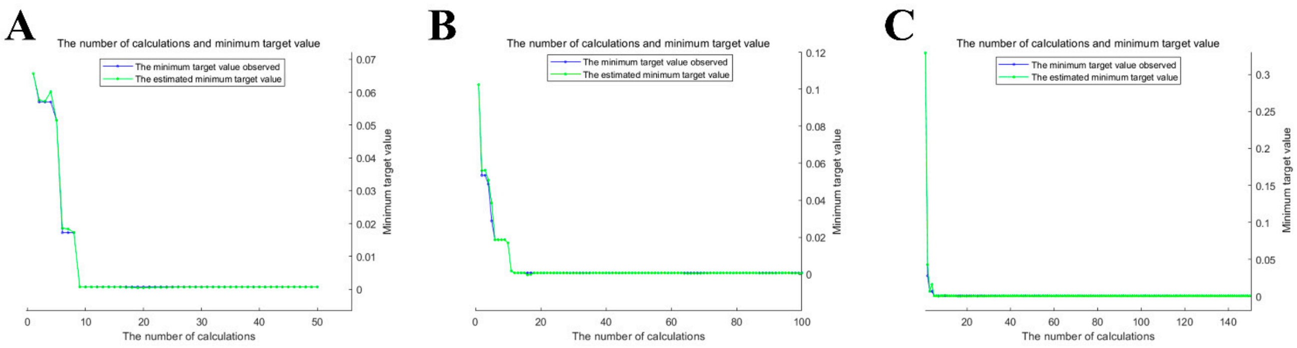

Figure S1: Convergence of the minimum target value with increasing calculations in Bayesian optimization: (A) 50 iterations, (B) 100 iterations, (C) 150 iterations.

References

1. Idrees, F.; Hou, J.; Cao, C.; Butt, F.K.; Shakir, I.; Tahir, M.; Idrees, F. Template-Free Synthesis of Highly Ordered 3D-Hollow Hierarchical Nb2O5 Superstructures as an Asymmetric Supercapacitor by Using Inorganic Electrolyte. *Electrochimica Acta* **2016**, *216*, 332–338, doi:10.1016/j.electacta.2016.09.031.
2. Huang, K.-J.; Zhang, J.-Z.; Shi, G.-W.; Liu, Y.-M. Hydrothermal Synthesis of Molybdenum Disulfide Nanosheets as Supercapacitors Electrode Material. *Electrochimica Acta* **2014**, *132*, 397–403, doi:10.1016/j.electacta.2014.04.007.
3. Tong, L.; Song, C.; Liu, Y.; Xing, R.; Sekar, K.; Liu, S. Open and Porous NiS2 Nanowrinkles Grown on Non-Stoichiometric MoOx Nanorods for High-Performance Alkaline Water Electrolysis and Supercapacitor. *International Journal of Hydrogen Energy* **2022**, *47*, 14404–14413, doi:10.1016/j.ijhydene.2022.02.190.
4. Wang, L.; Qin, J.; Bai, Z.; Hao, Y.; Li, W.; Wang, J.; Li, X. Core-Branched FeOOH-CNTs Composites for High-Performance Supercapacitors. *Materials Letters* **2022**, *322*, 132442, doi:10.1016/j.matlet.2022.132442.
5. Chen, C.; Liu, M.; Liu, Z.; Xie, M.; Wan, L.; Chen, J.; Zhang, Y.; Du, C.; Li, D. Design of Mesoporous Ni-Co Hydroxides Nanosheets Stabilized by BO2- for Pseudocapacitors with Superior Performance. *Journal of Colloid and Interface Science* **2022**, *614*, 66–74, doi:10.1016/j.jcis.2022.01.028.
6. Wang, C.; Qu, H.; Peng, T.; Mei, K.; Qiu, Y.; Lu, Y.; Luo, Y.; Yu, B. Large Scale α-Co(OH)2 Needle Arrays Grown on Carbon Nanotube Foams as Free Standing Electrodes for Supercapacitors. *Electrochimica Acta* **2016**, *191*, 133–141, doi:10.1016/j.electacta.2016.01.057.
7. Keshari, A.S.; Dubey, P. Rapid Microwave-Assisted vs. Hydrothermal Synthesis of Hierarchical Sheet-like NiO/NiMoO4 Hybrid Nanostructures for High Performance Extrinsic Pseudocapacitor Application. *Journal of Energy Storage* **2021**, *40*, 102629, doi:10.1016/j.est.2021.102629.
8. Xu, M.; Chen, L.; Zhou, W.; Liu, X.; Lu, D.; Zhang, M.; Shi, Z. Manganese Oxides In-Situ Grown on Carbon Sphere and Derived Different Crystal Structures as High-Performance Pseudocapacitor Electrode Material. *Journal of Alloys and Compounds* **2021**, *878*, 160384, doi:10.1016/j.jallcom.2021.160384.
9. Alex, J.; Rajkumar, S.; Ali, S.R.; Kunjumon, J.; Merlin, J.P.; Aravind, A.; Sajan, D.; Praveen, C.S. Charge Storage Mechanism and Pseudocapacitance Performance of NiO and Ce-Doped NiO Synthesized via Modified Combustion Technique. *Ceramics International* **2024**, *50*, 47317–47332, doi:10.1016/j.ceramint.2024.09.083.
10. Xie, Y.; Wang, Y.; Wang, Y.; Liu, Y.; Zhang, H.; Yang, R.; Marin, E.; Pezzotti, G.; Zhu, W. Effects of Ce Doping on Specific Capacitance of ZIF-8 Derived Carbon. *Materials Today Communications* **2024**, *41*, 110504, doi:10.1016/j.mtcomm.2024.110504.
11. Wang, H.-Y.; Li, D.-G.; Zhu, H.-L.; Qi, Y.-X.; Li, H.; Lun, N.; Bai, Y.-J. Mn3O4/Ni(OH)2 Nanocomposite as an Applicable Electrode Material for Pseudocapacitors. *Electrochimica Acta* **2017**, *249*, 155–165, doi:10.1016/j.electacta.2017.08.015.
12. Qian, J.; Wang, Y.; Chen, Z.; Liu, C.; Zhou, Y.; Yang, Y.; Song, Y.; Kong, B. Three Dimensional Mn3O4-CeO2/Holey-Graphene Hierarchical Architectures from Stem for High-Performance Asymmetric Supercapacitors. *Inorganic Chemistry Communications* **2019**, *104*, 8–13, doi:10.1016/j.inoche.2019.03.032.
13. Qian, J.; Wang, Y.; Chen, Z.; Liu, C.; Zhou, Y.; Yang, Y.; Song, Y.; Kong, B. Three Dimensional Mn3O4-CeO2/Holey-Graphene Hierarchical Architectures from Stem for High-Performance Asymmetric Supercapacitors. *Inorganic Chemistry Communications* **2019**, *104*, 8–13, doi:10.1016/j.inoche.2019.03.032.
14. Shinde, S.K.; Karade, S.S.; Maile, N.C.; Yadav, H.M.; Ghodake, G.S.; Jagadale, A.D.; Jalak, M.B.; Lee, D.S.; Kim, D.-Y. Synthesis of 3D Nanoflower-like Mesoporous NiCo2O4 N-Doped CNTs Nanocomposite for Solid-State Hybrid Supercapacitor; Efficient Material for the Positive Electrode. *Ceramics International* **2021**, *47*, 31650–31665, doi:10.1016/j.ceramint.2021.08.045.
15. Wang, Q.; Zou, Y.; Xiang, C.; Chu, H.; Zhang, H.; Xu, F.; Sun, L.; Tang, C. High-Performance Supercapacitor Based on V2O5/Carbon Nanotubes-Super Activated Carbon Ternary Composite. *Ceramics International* **2016**, *42*, 12129–12135, doi:10.1016/j.ceramint.2016.04.145.
16. Isacfranklin, M.; Yuvakkumar, R.; Ravi, G.; Thambidurai, M.; Nguyen, H.D.; Velauthapillai, D. SmNiO3/SWCNT Perovskite Composite for Hybrid Supercapacitor. *Journal of Energy Storage* **2023**, *68*, 107786, doi:10.1016/j.est.2023.107786.
17. Vidyadharan, B.; Aziz, R.A.; Misnon, I.I.; Anil Kumar, G.M.; Ismail, J.; Yusoff, M.M.; Jose, R. High Energy and Power Density Asymmetric Supercapacitors Using Electrospun Cobalt Oxide Nanowire Anode. *Journal of Power Sources* **2014**, *270*, 526–535, doi:10.1016/j.jpowsour.2014.07.134.
18. Denis, D.K.; Sun, X.; Zhang, J.; Wang, Y.; Hou, L.; Li, J.; Yuan, C. Solid Solution Engineering of Co–Ni-Based Ternary Molybdate Nanorods toward Hybrid Supercapacitors and Lithium-Ion Batteries as High-Performance Electrodes. *ACS Appl. Energy Mater.* **2020**, *3*, 3955–3965, doi:10.1021/acsaem.0c00353.
19. Naseem, K.; Ali, Z.; Chen, P.; Tahir, A.; Qin, F.; Fayyaz, A.; Albaqami, M.D.; Mohammad, S.;

Akkinepally, B.; Ali, S.; et al. Supercapacitive Behavior and Energy Storage Properties of Molybdenum Carbide Ceramics Synthesized via Ball Milling Technique. *Ceramics International* **2024**, *50*, 9572–9580, doi:10.1016/j.ceramint.2023.12.276.
20. Lee, Y.-S.; Selvaraj, A.R.; Kostoglou, N.; Rebholz, C.; Rajendiran, R.; Raman, V.; Kim, H.; Rajesh, J.A.; Nagulapati, V.M.; Oh, T.H.; et al. Asymmetric Supercapacitors Based on Biomass-Derived Porous Activated Carbon (PAC)/1D Manganese Oxide (MnO2) Electrodes with High Power and Energy Densities. *Materials Science and Engineering: B* **2024**, *304*, 117368, doi:10.1016/j.mseb.2024.117368.
21. Zhao, Z.; Zhang, M.; Jin, Y.; Han, Y.; Sun, X.; An, C.J. Designing Intimately Interconnected Neuron-Shaped Nickel-Copper(I) Sulfide Nanocomposites as Cathode Material for Supercapacitors. *Journal of Energy Storage* **2024**, *89*, 111749, doi:10.1016/j.est.2024.111749.
22. Sadiq, M.; Islam, M.U. Tailoring the Electrochemical Performance of Novel BaS and Their N-rGO Composites for Developing next-Generation Pseudocapacitor Electrodes. *Journal of Energy Storage* **2024**, *100*, 113525, doi:10.1016/j.est.2024.113525.
23. Hassan, N.U.; Jabeen, N.; Younas, W.; Ahmed, F.; Hussain, A.; Asif, S.U.; Alghamdi, M.M.; Naveed, M. Efficient Hybrid Supercapacitor Performance Enabled by Large Surface Area of 2D Mesoporous Zinc Sulfide Nano-Sheets Synthesized via Microwaves. *Journal of Electroanalytical Chemistry* **2024**, *975*, 118794, doi:10.1016/j.jelechem.2024.118794.
24. Pitcheri, R.; Mooni, S.P.; Radhalayam, D.; Nora, M.; Roy, S.; Al-Zahrani, F.A.M.; Suneetha, M. Effect of Ce-Doping on the Structural, Morphological, and Electrochemical Features of Co3O4 Nanoparticles Synthesized by Solution Combustion Method for Battery-Type Supercapacitors. *Ceramics International* **2024**, *50*, 50504–50515, doi:10.1016/j.ceramint.2024.09.396.
25. Ranjithkumar, R.; Ho Youk, J. Nitrogen-Doped Mesoporous Carbon Spheres Decorated with NiCo Alloy Nanoparticles for High-Performance Electrochemical Supercapacitors. *Journal of Electroanalytical Chemistry* **2024**, *960*, 118183, doi:10.1016/j.jelechem.2024.118183.
26. Ali, F.; Khalid, N.R. Effect of Calcination Temperature on Structural, Morphological and Electrochemical Properties of Sn Doped Co3O4 Nanorods. *Ceramics International* **2020**, *46*, 24137–24146, doi:10.1016/j.ceramint.2020.06.193.
27. Liu, S.; Cai, Y.; Zhao, X.; Liang, Y.; Zheng, M.; Hu, H.; Dong, H.; Jiang, S.; Liu, Y.; Xiao, Y. Sulfur-Doped Nanoporous Carbon Spheres with Ultrahigh Specific Surface Area and High Electrochemical Activity for Supercapacitor. *Journal of Power Sources* **2017**, *360*, 373–382, doi:10.1016/j.jpowsour.2017.06.029.
28. Muthu, D.; Vargheese, S.; Haldorai, Y.; Rajendra Kumar, R.T. NiMoO4/Reduced Graphene Oxide Composite as an Electrode Material for Hybrid Supercapacitor. *Materials Science in Semiconductor Processing* **2021**, *135*, 106078, doi:10.1016/j.mssp.2021.106078.
29. Zhang, Y.; Zhou, W.; Yu, H.; Feng, T.; Pu, Y.; Liu, H.; Xiao, W.; Tian, L. Self-Templated Synthesis of Nickel Silicate Hydroxide/Reduced Graphene Oxide Composite Hollow Microspheres as Highly Stable Supercapacitor Electrode Material. *Nanoscale Res Lett* **2017**, *12*, 325, doi:10.1186/s11671-017-2094-9.
30. Tong, J.; Zhang, H.; Gu, J.; Li, L.; Ma, C.; Zhao, J.; Wang, C. Poly(Ethylene Glycol)-Block-Poly(Propylene Glycol)-Block-Poly(Ethylene Glycol)-Assisted Synthesis of Graphene/Polyaniline Composites as High-Performance Supercapacitor Electrodes. *J Mater Sci* **2016**, *51*, 1966–1977, doi:10.1007/s10853-015-9506-y.
31. Zhao, K.; Lyu, K.; Liu, S.; Gan, Q.; He, Z.; Zhou, Z. Ordered Porous Mn3O4@N-Doped Carbon/Graphene Hybrids Derived from Metal–Organic Frameworks for Supercapacitor Electrodes. *J Mater Sci* **2017**, *52*, 446–457, doi:10.1007/s10853-016-0344-3.
32. Tan, Y.; Xu, C.; Chen, G.; Liu, Z.; Ma, M.; Xie, Q.; Zheng, N.; Yao, S. Synthesis of Ultrathin Nitrogen-Doped Graphitic Carbon Nanocages as Advanced Electrode Materials for Supercapacitor. *ACS Appl. Mater. Interfaces* **2013**, *5*, 2241–2248, doi:10.1021/am400001g.
33. Chen, H.; Zhou, S.; Wu, L. Porous Nickel Hydroxide–Manganese Dioxide-Reduced Graphene Oxide Ternary Hybrid Spheres as Excellent Supercapacitor Electrode Materials. *ACS Appl. Mater. Interfaces* **2014**, *6*, 8621–8630, doi:10.1021/am5014375.
34. Xu, J.; Zhou, X.; Chen, M.; Shi, S.; Cao, Y. Preparing Hierarchical Porous Carbon Aerogels Based on Enzymatic Hydrolysis Lignin through Ambient Drying for Supercapacitor Electrodes. *Microporous and Mesoporous Materials* **2018**, *265*, 258–265, doi:10.1016/j.micromeso.2018.02.024.
35. Gong, Y.; Li, D.; Luo, C.; Fu, Q.; Pan, C. Highly Porous Graphitic Biomass Carbon as Advanced Electrode Materials for Supercapacitors. *Green Chem.* **2017**, *19*, 4132–4140, doi:10.1039/C7GC01681F.
36. Shi, M.; Xin, Y.; Chen, X.; Zou, K.; Jing, W.; Sun, J.; Chen, Y.; Liu, Y. Coal-Derived Porous Activated Carbon with Ultrahigh Specific Surface Area and Excellent Electrochemical Performance for

Supercapacitors. *Journal of Alloys and Compounds* **2021**, *859*, 157856, doi:10.1016/j.jallcom.2020.157856.
37. Su, X.-L.; Li, S.-H.; Jiang, S.; Peng, Z.-K.; Guan, X.-X.; Zheng, X.-C. Superior Capacitive Behavior of Porous Activated Carbon Tubes Derived from Biomass Waste-Cotonier Strobili Fibers. *Advanced Powder Technology* **2018**, *29*, 2097–2107, doi:10.1016/j.apt.2018.05.018.
38. Asif, S.U.; Haider, A.A.; Zhang, S.; Chen, X.; Wang, S. Designing Fluorite Structured Samarium Doped Bismuth Yttrium Oxide Perovskites Based High-Performance Electrodes for Advanced Supercapacitor Applications. *Journal of Rare Earths* **2025**, doi:10.1016/j.jre.2025.05.009.
39. Li, W.; Yang, X.; Yuan, C.; Wang, X.; Huo, X.; Ye, Y.; Qian, Z.; Qin, Z. Optimizing the Degree of Structural Disorder in Coal-Based Porous Carbon for Enhancing Supercapacitor Capacitance Performance. *Fuel* **2026**, *404*, 136378, doi:10.1016/j.fuel.2025.136378.
40. Zhang, Y.; Xu, J.; Lu, S.; Li, H.; Yonar, T.; Hua, Q.; Liu, T.; Zhang, Y. Engineering Few-Layer MoS2 and rGO Heterostructure Composites for High-Performance Supercapacitors. *Adv Compos Hybrid Mater* **2025**, *8*, 108, doi:10.1007/s42114-024-01159-z.
41. Pang, Z.; Duan, J.; Zhao, Y.; Tang, Q.; He, B.; Yu, L. A Ceramic NiO/ZrO2 Separator for High-Temperature Supercapacitor up to 140 °C. *Journal of Power Sources* **2018**, *400*, 126–134, doi:10.1016/j.jpowsour.2018.08.008.
